# Hosts and Heterologous Expression Strategies of Recombinant Toxins for Therapeutic Purposes

**DOI:** 10.3390/toxins15120699

**Published:** 2023-12-13

**Authors:** Luana di Leandro, Martina Colasante, Giuseppina Pitari, Rodolfo Ippoliti

**Affiliations:** Department of Life, Health and Environmental Sciences, University of L’Aquila, 67100 L’Aquila, Italy; luana.dileandro@univaq.it (L.d.L.); martina.colasante@student.univaq.it (M.C.); giuseppina.pitari@univaq.it (G.P.)

**Keywords:** recombinant toxins, immunotoxins, cancer research, host selection, expression strategies

## Abstract

The production of therapeutic recombinant toxins requires careful host cell selection. Bacteria, yeast, and mammalian cells are common choices, but no universal solution exists. Achieving the delicate balance in toxin production is crucial due to potential self-intoxication. Recombinant toxins from various sources find applications in antimicrobials, biotechnology, cancer drugs, and vaccines. “Toxin-based therapy” targets diseased cells using three strategies. Targeted cancer therapy, like antibody–toxin conjugates, fusion toxins, or “suicide gene therapy”, can selectively eliminate cancer cells, leaving healthy cells unharmed. Notable toxins from various biological sources may be used as full-length toxins, as plant (saporin) or animal (melittin) toxins, or as isolated domains that are typical of bacterial toxins, including Pseudomonas Exotoxin A (PE) and diphtheria toxin (DT). This paper outlines toxin expression methods and system advantages and disadvantages, emphasizing host cell selection’s critical role.

## 1. Introduction

The commercial production of recombinant proteins for therapeutic purposes involves the utilization of various hosts, with the most common choices being bacteria, yeasts, and mammalian cell lines. However, identifying the most suitable host cell is arguably the most critical step in the entire production process. There are no universally ideal organisms capable of safely and efficiently producing all types of proteins. Therefore, the selection of an expression system must be carefully considered based on the characteristics of the final product.

*Escherichia coli* is used for the recombinant production of at least 30% of the therapeutic proteins currently approved and has been—due to its well-known genetics, high rate of growth, and, in many cases, high yields—considered a favored platform in the biotech sector for its expression of proteins. For *E. coli* systems, there is a plethora of knowledge and extensive tools, such as vectors suitable for expression, selected strains, technologies endowed with fermentation, and strategies suitable for increasing protein folding, that are ideally developed in industrial applications. Recent advances in complex protein expression, such as full-length antibodies in non-glycosylated form, the engineering of new strains, N-glycosylation in bacterial organisms, and finally, cell-free systems, suggest that complex proteins and humanized glycoproteins can be produced in *E. coli* and that several strains and a lot of expression vectors have been engineered, such as the BL21(DE3)-pLysS for handling toxic proteins. *E. coli* can grow rapidly and produce high-density cultures using quite inexpensive culture media, allowing, in many cases, for high yields of the protein of interest.

Eukaryotic cells can be utilized as an alternative to prokaryotic cells for the expression in those cases requiring the strict control of the folding and assembly of multi-subunit proteins and the correct formation of disulfides since they have the required molecular, genetic, and metabolic characteristics. Because yeast cells are recognized as safe (GRAS) organisms, they are advantageous host organisms for the biopharmaceutical manufacturing of therapeutic recombinant proteins. The classic baker’s yeast *Saccharomyces cerevisiae* is the most-characterized eukaryotic and extensively utilized host for recombinant therapeutic proteins, but other yeast species, such as *Pichia pastoris, Kluyveromyces lactis, Hansenula polymorpha*, *Yarrowia lipolytica*, and *Schizosaccharomyces pombe*, have also been established as efficient hosts for their production.

Many mammalian cell lines have been used to express proteins; the most widely used ones are CHO (Chinese hamster ovary) and HEK 293 (human embryonic kidney), but also, cell lines such as J558L and Sp2/0 from myeloma, Vero cells, mouse L-cells, and baby hamster kidney (BHK) cells are frequently used as hosts to create stable transfectants. These cells are used to express a variety of heterologous proteins, from viral structural proteins to bioactive peptides, even though their use in large-scale industrial production has been limited by high cost, complex technology, and potential for animal virus contamination.

Baculovirus/insect systems are often used to obtain high levels of expression of recombinant glycoprotein to produce vaccines and used for gene therapy because this latter is safe for vertebrates, but continuous expression via fermentation is not possible [1], thus limiting its application at productive levels.

One of the key challenges in producing recombinant proteins is achieving a delicate balance to obtain properly folded proteins with the precise disulfide pattern (if present). This is particularly important in toxin production, given the inherent paradox where an increase in toxin production may poison host cells, compromising their viability and, consequently, the production of the toxin itself. Potential self-intoxication must also be considered when identifying the most suitable expression system.

Toxins are derived from diverse biological sources, such as bacteria, yeast, scorpions, snakes, spiders, and other species, and are widely used in a variety of applications: (i) antimicrobial agents for medicinal use, (ii) components for the production of anticancer drugs and the treatment of neurological diseases [2], (iii) vaccine development, and (iv) in some cases for the production of GMO plants (corn, maize, papaya, soybeans, and tomato) resistant to certain insect pests in biotechnological industries [3].

In particular, the term “toxin-based therapy” denotes a novel area of clinical research where toxic proteins, or their gene sequences, are employed in various ways to target diseased cells and tissues. Three targeting systems have been studied in toxin-based therapy: (i) antibody-targeted toxins (immunotoxins), (ii) ligand-targeted toxins (fusions), and (iii) ligand (peptide or nucleic acid)-targeted toxin-based suicide gene therapy.

Due to the limited therapeutic window and off-target effects of undirected anti-cancer cytotoxins, targeted cancer therapy is often preferred over systemically effective cytotoxic medications. Promisingly, antibody–toxin conjugates, such as chemically linked (ITs) and recombinant immunotoxins (RITs), represent a significant class of targeted anti-cancer therapeutics. These agents selectively eliminate cancerous cells by targeting cancer-associated antigens, leaving healthy cells unaffected. RITs, in contrast to a well-known class of targeted therapeutic agents called ADCs (antibody–drug conjugates) that carry a synthetic drug, incorporate a protein toxin or its domain that is typically not of human origin as their cytotoxic component. It is possible to express that component directly, either on its own or as fusion proteins, in host cells.

The most used toxin domains are represented by recombinant forms of bacterial toxins like diphtheria toxin (DT) and Pseudomonas Exotoxin A (PE), both of which inhibit the eukaryotic elongation factor 2 (eEF2). Additionally, plant toxins that target eukaryotic 28S ribosomal RNA, such as ricin and saporin, have found widespread use.

This paper deals with the methods of expressing some of the most studied toxic proteins produced as recombinants in bacteria, yeast, insect cells, or mammalian cells for therapeutic purposes (i.e., the production of immunotoxins or fusion toxins). It also provides an overview of the primary advantages and disadvantages of these various systems for toxin manufacturing.

## 2. Bacterial Toxins

### 2.1. Diphtheria Toxin

Diphtheria toxin (DT) is a 62 kDa protein produced as a secretory toxin via the Gram-positive bacterium *Corynebacterium diphtheriae* [4,5]. DT consists of two fragments referred to as A and B. Fragment B binds to the cell surface, recognizing a specific receptor (heparin-binding EGF-like growth factor, HB-EGF) and then allows the transfer of fragment A into the cell. Diphtheria toxin fragment A (DTA) enzymatically catalyzes ADP-dependent ribosylation of a histidine residue in eukaryotic elongation factor 2 (EF-2); this activity makes diphtheria toxin (DT) poisonous, leading to cell death due to the inhibition of protein synthesis [6,7].

**Figure 1:**
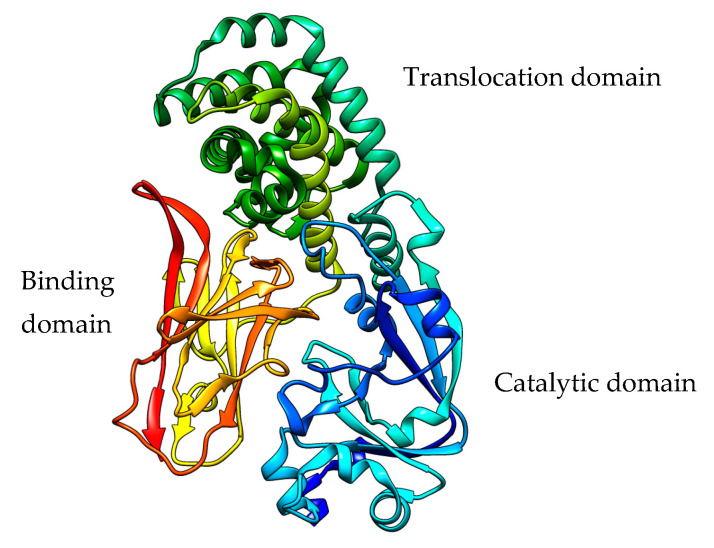
The crystal structure of diphtheria toxin (PDB code: 1F0L). DT consists of three structural domains: catalytic domain (blue), translocation domain (green), and receptor binding domain (red and yellow). The structure was created using the UCSF Chimera Software (1.17.3).

In *Corynebacterium diphtheriae,* its natural host, DT is produced in an inactive form consisting of three different domains (A, B, and T): an N-terminal signal sequence is present, which, once removed, activates further processing with proteolytic separation of the A and B domains (that become subunits) still linked with a disulfide bridge and secreted into the extracellular medium [8]. The extracellularly released toxin can bind to its receptor via the B subunit and be internalized through receptor-mediated endocytosis. In acidic conditions within the endosomes, the T (translocation) domain passes through the endosomal membrane, allowing the passage of the active A chain into the cytosolic lumen, where reduction in the disulfide bond releases the A chain, causing it to exert its toxicity (Figure 1).

**Figure 2:**
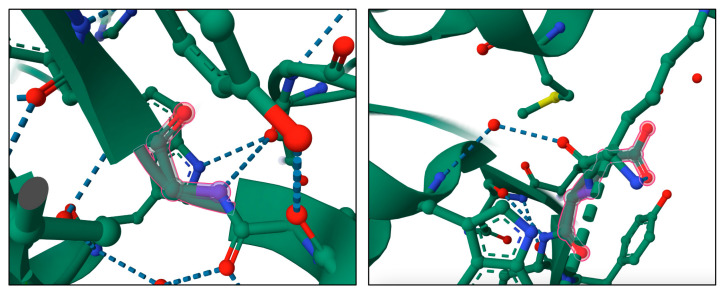
A single amino acid mutation, G52E, with glycine at position 52 (**left**) replaced by glutamic acid (**right**) in CRM197 (PDB code: 5I82). The structure of the protein is green, the amino acids at the mutation site are opaque and the outline is red. The structure was created using the UCSF Chimera Software (1.17.3).

Before the use of DT as a toxic component of immunotoxins, a detailed study of the structure–function correlation of DT allowed the elimination of toxic enzyme activity, thus making it suitable for vaccine development. In 1971, Uchida et al. demonstrated that upon mutagenesis of βtox+ corynebacteriophage with the use of nitrosoguanidine, a number of phages encoding non-toxic proteins could be isolated [9]; these were named cross-reactive material (CRM) and were demonstrated to be immunologically related to DT [6]. The most promising results were shown by cross-reacting material 197 (CRM197; Figure 2). It contains a single amino acid mutation, with glycine at position 52 replaced by glutamic acid (G52E), resulting in a significantly reduced ability to bind NAD and, thus, a lack of toxicity, being about 106 times less toxic than DT [6,10]. Another mutant, CRM176, involves an aspartate-to-glycine substitution at position 128. The toxic activity of this mutant is approximately 10% of DT. Over the years, however, CRM197 has been much more widely used [11].

CRM197′s initial use was as a carrier protein in conjugate vaccines, and it is actually present in several marketed vaccines such as Menveo, Menjugate, and Vaxneuvance vaccines [8,12,13]. CRM197 can produce a T-lymphocyte-dependent immunogenic response against otherwise poorly immunogenic polysaccharides. Furthermore, CRM197 does not need chemical detoxification, and its T-helper epitopes are nicely conserved, thus giving rise to superior effectiveness as a carrier compared to chemically inactivated DT. Besides its role as a vaccine adjuvant, CRM197 gained interest for its potential antitumor activity, which is correlated to the recognition and binding of the soluble form of HB-EGF, a marker that is highly expressed in several human tumors. Moreover, CRM197 is considered a safe drug against atherosclerosis [11,14,15].

Efforts have been made to industrially produce soluble CRM197 in different host organisms. Traditionally, it has been produced using *C. diphtheriae* mutant strains to recover CRM197 from the culture supernatant to allow its purification [16]. However, yields are usually in the range of 100–150 mg/L, which is relatively low. Additionally, the expression of both CRM197 and DT itself requires very specific conditions, including low iron concentration, temperature, agitation, and aeration [17], making industrial cultivation of *C. diphtheriae* quite challenging. For this reason, several alternative, inexpensive, and high-yield expression systems were tested over time, including *E. coli*, *Bacillus subtilis*, and *Pseudomonas fluorescens* [18]. *P. fluorescens* has been used to produce a commercial form of CRM197 (Pfenex), giving yields of 1–2 g/L.

Spheroplasts of *Saccharomyces cerevisiae* and mammalian cells present many limitations as expression hosts for CRM197, as it has been observed that the latter induces high cytotoxicity [19].

#### Expression Hosts for DT or Its Mutants

- DT expression in Bacteria

The DT full gene could be cloned using particular restrictions and following the high-level containment rule, but DNA fragments encoding certain non-toxic or hypo-toxic fragments of the protein can be more easily cloned and expressed in *E. coli* [20]. In the literature, however, there are several reports of the DT full gene cloning and expression in *E. coli*, most of which are for vaccine production; recently, whole DT and its fragment B genes from the strain *Corynebacterium diphtheriae*, originating from the Corynebacterium diphtheriae Park William strain, were expressed in *E. coli*. This approach aims to address the significant drawbacks associated with traditional anti-diphtheria vaccines.

The authors demonstrated that the proteins were expressed in discrete quantities (0.9–1.12 mg/mL) under batch culture conditions implemented by fed-batch, improving biomasses with the addition of glucose and yeast extract as carbon sources [21]. A mutant, full-length form of diphtheria toxin (Glu148Ser), 800-fold less cytotoxic than a wild-type toxin, was cloned and expressed into *E. coli K-12* under BL-1EK-1 conditions, and the protein has been recovered from the periplasmic extracts [22].

Due to the toxin’s high cytotoxicity, many attempts have been made to bind its fragments to various ligands to focus its toxicity on specific cells. The toxic moiety of these hybrid molecules was usually used in the diphtheria toxin fragment A, Cross-reacting material-45 (CRM45, 45-kDa) tox nonsense mutant, or the whole toxin are used AS toxic moiety of these hybrid molecules. In 1980, Gilliland et al. made one of the initial attempts to employ diphtheria toxin in the development of Paul Ehrlich’s “magic bullets”. This involved the conjugation of ricin A chain and diphtheria toxin Fragment A to monoclonal antibodies designed to target a cell surface antigen present in colorectal carcinoma cells [23]. Recombinant immunotoxins for cancer treatment have then been designed using truncated forms of DT [24]. The genetic replacement of the native DT receptor-binding domain with growth factors, cytokines, cell-penetrating peptides, and other specific ligands recognizing cancer antigens has led to the creation of fusion proteins that maintain the activities and functions of their individual components [25]. Some of DT truncated form immunotoxins and fusion proteins expressed recombinantly used for cancer therapies are listed in the following Table 1.

As shown above, most DT immunotoxins used for clinical trials were produced in *E. coli* and were harvested from insoluble inclusion bodies after extensive washing to remove endotoxins, solubilization, and denaturation steps. In the case of DT388-GM-CSF, the immunotoxin is recovered from the cytoplasm and simply purified via affinity chromatography [30]. A diphtheria toxin-based recombinant fusion toxin (Ontak) has been approved by the FDA for the treatment of human CD25^+^ cutaneous T-cell lymphoma (CTCL). It was marketed in the United States from 1999 to 2014, but issues about the presence of heterogeneous molecular weight protein aggregates, excess residual detergent, and excess residual DNA in the final formulation led the FDA to put Ontak^®^ on clinical hold. A new formulation named E7777 has the same Ontak amino acid sequence but improved purity and bioactivity. The newly developed E7777 expression strategy led to obtaining an increase in immunotoxin monomer species, with a parallel decrease in levels of protein misfolding and aggregation with ~1.5–2 times increase in specific bioactivity when tested in non-clinical assays, and it is actually in a Phase III clinical trial (ClinicalTrials.gov identifier NCT01871727, 6 December 2021 [47]). Changing the expression species could also represent an alternative strategy to solve the aggregation problems observed for Ontak^TM^ as obtained for s-DAB-IL2(V6A) [29] in *C. diphteriae* or for DAB389IL2IL2 in *P. pastoris* [28]. In the first case, the structural gene for Ontak^®^, DAB389IL-2, has been cloned in an *E. coli*/*C. diphtheriae* shuttle vector. In *C. diphtheriae*, the tox operon is composed of a *tox* promoter/operator (*tox*PO) upstream of the DT encoding gene, whose expression is regulated via the diphtheria toxin repressor (DtxR) that is a regulatory protein using Fe^2+^ as co-repressor. In the presence of divalent transition metal cations (mainly Fe^+2^, but also Co^+2^, Mn^+2^, Ni^+2^, and Cd^+2^), DtxR changes its structures to form dimers. Two DtxR dimers interact with opposite faces of toxO, shielding the “−10” sequence of toxP, thus finally repressing transcription [48]. To get the fusion toxin secreted into the culture medium, the authors modified the immunotoxin construct by reintroducing the native *tox* signal sequence to make expression constitutive in a culture medium with high iron content and also incorporated mutations inside the palindromic *tox* operator at the level of the downstream half [29].

Uchida et al. [9] isolated corynebacteriophage mutant *C. diphtheriae* lysogens that secreted non-toxic proteins serologically related to diphtheria toxin. The isolation of CRMs resulting from both nonsense (e.g., CRM45) and missense mutations (e.g., CRM197) determined the N- to C-terminal orientation of the toxin and clarified its splicing.

The expression in *E. coli* of DT or CRM197, an inactive variant of DT used as a delivery system [8,12] to immunize, eliminates the need for BSL-2 containment requirements, which are essential when working with *C. diphtheriae*. Over the years, various research groups have employed different strategies to achieve good protein yields, including purifying the protein from inclusion bodies or periplasm, as well as exploring methods to obtain the protein directly in the culture medium.

The production of both DT and CRM197 in *E. coli* is hindered by the formation of inclusion bodies, leading to the precipitation of the heterologous protein within them. Working with inclusion bodies necessitates a critical protein folding step after purification, which, while resulting in high yields, also produces increased downstream processing costs [49].

Traditionally, highly concentrated denaturing solubilizing agents like urea or guanidine hydrochloride (GdnHCl) are used to solubilize inclusion bodies [50,51]. When proteins contain multiple cysteine residues, β-mercaptoethanol or dithiothreitol is added to prevent the formation of incorrect disulfide bonds [52]. However, using high concentrations of chaotropic reagents for solubilization can lead to the complete destruction of protein structure, often resulting in protein aggregation and precipitation, especially when dealing with proteins containing multiple cysteine residues [53]. CRM197, which possesses two intramolecular disulfide bonds between positions 186:201 and 461:471 [54], has led several research groups to explore solubilization methods with non-denaturing agents.

Ah-Reum Park and colleagues [54] utilized N-lauroylsarkosine to recover active CRM197 efficiently from an insoluble pellet. They demonstrated a remarkable >80% yield, a significant improvement compared to yields obtained with denaturing agents. Moreover, the secretion of CRM197 into the periplasm has been explored as a cost-effective approach to protein recovery.

Given that the folding and activity of CRM197 involve two disulfide bonds, expression in the periplasm presents a compelling alternative. The periplasm creates an oxidative environment equipped with a specialized enzyme system (Dsb) responsible for catalyzing bond formation [55]. Other advantages include reduced proteolysis, N-terminal authenticity following cleavage of the signal peptide, and higher purity of the recombinant protein [56,57].

Sec and SRP (signal recognition particle) are the two commonly used systems for secretion and periplasmic delivery of recombinant proteins, sharing the same translocator, SecYEG. However, they target proteins to the secretion machinery differently. Translocation through the SRP pathway occurs co-translationally, while the SEC pathway induces translocation of unfolded proteins as a post-translational event.

By using the ssFlgI signal sequence, Goffin and colleagues achieved substantial CRM197 production (>3 g/L) in the periplasm of optimized high-density cultures, a 20-fold increase compared to the typical process with *C. diphtheriae*. Significantly, the yield was substantial, and CRM197 demonstrated proper folding with disulfide bonds in their correct positions. Additionally, the N-terminal matched precisely with the CRM197 sequence from C. diphtheriae, confirming the effective removal of the signal peptide.

ssFlgI is the signal sequence that, when combined with CRM197, produces the best combination of yield and secretion [58].

The efficient formation of the two disulfide bonds is challenging in the reducing cytoplasmic environment, often leading to partial or total insolubility of the protein. In some cases, despite codon optimization, CRM197 production is minimal or absent [59,60]. One potential strategy involves host cell engineering, including mutants with a reduced environment [61] or overexpression of chaperones [60].

An alternative method to produce soluble CRM197 involves the co-expression of disulfide isomerase (PDI) and sulfhydryl oxidase (SOX). Because the two disulfide bonds typically cannot form in the reducing cytoplasmic environment, co-expression of SOX and PDI has been found to improve soluble CRM197 production. This method yields approximately 10 percent of insoluble CRM197 production in equivalent small-scale cultures. SOX and PDI both control the formation of intra-protein disulfide bonds and can be considered checkpoints for the tertiary structure of the produced protein [62].

A recombinant immunotoxin named Tagraxofusp (Elzonris^®^), composed of human interleukin-3 fused to a truncated diphtheria toxin, was approved by the FDA in December 2018 and was authorized for the treatment of Blastic Plasmacytoid Dendritic Cell Neoplasm (BPDCN) in both adult and pediatric patients. This immunotoxin, named DT388-IL3, was first produced in *E. coli* in 2003. The first 388 amino acid residues of DT were fused to human interleukin-3 with a fused HM linker. The protein was expressed in *E. coli* BLR (DE3) and purified from inclusion bodies following extraction with guanidine hydrochloride and dithioerythritol; the resulting recombinant protein was folded in buffer with arginine and oxidized glutathione [31].

- DT Yeast Expression in Biological Research

Recombinant DT-based IT fusions were effectively expressed in *P. pastoris*, particularly in the GS115 strain. This strain was found to exhibit a high level of resistance to the bacterial toxin [63].

Researchers have harnessed *P. pastoris* to produce diverse DT fusion proteins. These include one with an interleukin-2 (IL-2) fusion to target CD25+ cells and another with DT fused to two single-chain variable fragment (scFv) tandem molecules responsible for binding to T lymphocytes [63,64].

In 2021, Aw et al. achieved industrial-scale production of CRM197, a secreted protein in *P. pastoris*, to be used in glycoconjugate vaccine development against typhoid [52]. The strain CBS7435 (ATCC 76723) and the vector PD912-AK were utilized, resulting in a yield of 113 mg/L after downstream processing. Successful yields have also been obtained using the AOX1 promoter or a constitutive GAP promoter [63,64,65]. 

Lowering the temperature to 15 °C has been shown to boost immunotoxin production by 50% [34]. Despite slightly lower yields, *P. pastoris*’s ability to secrete proteins directly into the supernatant positions it as a viable alternative to traditional hosts, surpassing yields obtained with *Bacillus subtilis* and comparable to those achieved using *Corynebacterium diphtheriae* [16,66].

The antiporcine CD3 recombinant immunotoxin A-dmDT390biscFv was expressed in *P. pastoris,* and after two-step purification, the obtained purification yield was ~13 mg per liter with 95% purity [67].

The ease of downstream processing and the use of a Generally Recognized As Safe (GRAS) organism with a rich and easily accessible medium make *P. pastoris* an extremely intriguing platform for further exploration [7] of DT-based immuno- or fusion toxins, taking into account all the great experience acquired for CRM197 expression.

- Adenovirus and Lentivirus in Gene Therapy with DT Gene

In the realm of gene therapy, adenoviral vectors stand out for their efficiency in gene transport compared to plasmid vectors. They are renowned for their safety and high expression efficiency [68,69]. These vectors, which frequently carry suicide genes, have been used in clinical trials and animal models to treat different types of cancer [70,71].

Notably, in the presence of exogenous testosterone, a PSA promoter-driven DTA gene sequence in an adenoviral vector displayed therapeutic benefits for prostate cancer cells [72]. Recent advancements have led to the development of adenoviral vectors with attenuated forms of DTA, such as DTA176 and DTA197. DTA197 shows promise in adenoviral gene therapy when controlled with the survival promoter [73]. DTA197 shows dose-related effects and has the potential to be used as a suicide gene in cancer gene therapy when it is under the control of the HSP promoter in plasmids [74].

DTA has also found utility in other therapeutic systems targeting HIV. For instance, a non-integrative, lentiviral vector, Rev-dependent, and encoding DTA and human TRAF6 have been used to target HIV reservoirs. Expression of this vector relies entirely on the presence of Rev, a protein expressed only in HIV-infected cells [75].

For use in in vitro negative selection techniques, scientists have designed another lentiviral vector expressing DTA controlled with the strong CMV promoter. This vector could be used in strategies that use a screening made via CRISPR/Cas9 to identify cell resistance to lentiviral vector infection, screen mutagenized envelope glycoproteins for cell type compatibility, or find envelope glycoprotein receptors and co-receptors that are not yet identified. Researchers engineered producer cells that were DTA-resistant and target cells using CRISPR/Cas9-mediated DPH1 knockout, a gene involved in the synthesis of diphthamide, the target of DTA’s catalytic activity, in order to enable abundant production of the DTA transgene in lentiviruses [76].

These advancements in adenoviral and lentiviral vector technology hold significant promise in the fields of gene therapy and targeted therapeutic interventions.

- DT expression in tobacco

Plant chloroplast gene expression offers the opportunity to increase the production of particular target proteins. In recent years, genetically modified plants have played a pivotal role in the production of various recombinant biopharmaceuticals, as reviewed by Daniell H. in 2006 [77]. Notably, these advancements have been particularly significant in the development of safe and cost-effective vaccines. A variety of plant species, including alfalfa as noted by Dong JL et al. in 2005, potato by Mason HS et al. in 2006, carrot by Marquet-Blouin E et al. in 2003, and Rosales-Mendoza S et al. in 2007, tomato as studied by Sandhu JS et al. in 2010, and tobacco as explored by Liu HL et al. in 2005 and Zhang H et al. in 2006, have been utilized in these endeavors [78,79,80,81,82,83,84].

One instance of particular interest is the production of diphtheria, pertussis, and tetanus (DPT) vaccines. This vaccine is widely administered to infants and children worldwide, and its efficacy is well-established. However, the conventional production process involves the purification of recombinant proteins from three different bacteria, incurring substantial costs. Efforts have been undertaken to create a multi-component recombinant DPT vaccine, as detailed by Soria-Guerra, R.E. et al. in 2009 [85].

An especially fascinating approach entails introducing and expressing a fusion protein that combines immune-protective epitopes derived from the exotoxins of *Clostridium tetani*, *Corynebacterium diphtheriae*, and *Bordetella pertussis* within tobacco chloroplasts, as detailed by Soria-Guerra, R.E. et al. in 2009 [85].

- DT expression in Mammalian cells

Eukaryotic cells are more likely to release highly active, properly folded proteins but are usually very sensitive to the toxin’s catalytic activity. The problem of the high toxicity of diphtheria toxin can be overcome by the presence of some mutations that can confer various degrees of resistance to mammalian cells for the reduced uptake or processing of the toxin. Only one type of mutation carries complete resistance to DT. Several research groups have found that inhibiting various proteins involved in diphthamide biosynthesis and DTA resistance can be achieved either through the expression of a dominant negative protein or through mutagenesis without affecting cellular viability. According to Gupta and Siminovitch (1978), Kohno and Uchida (1987), and Foley et al. (1992, 1995), this mutation modifies the structure of EF-2 and prevents the post-translational addition of the diphthamide structure; even when homozygous for the mutation, Chinese hamster ovary (CHO) cells with such mutations are very resistant to DT [86,87,88,89]. A further example is given with the human embryonic kidney cell line 293 transfected with the SV40 T antigen (293T) [90]. This line is particularly suitable for transient expression assays because it is readily transfected at high efficiency, and the SV40 T antigen replicates plasmids containing the SV40 origin. In work published in 2005 [43], this cell line was mutated, and clones that were highly resistant to DT were isolated and used to express DAB389-IL7, a very potent fusion toxin composed of the catalytic and transmembrane domains of diphtheria toxin fused to interleukin 7.

### 2.2. Pseudomonas aeruginosa Exotoxin A

Encounters with the opportunistic human pathogen *Pseudomonas aeruginosa*, an aerobic, Gram-negative bacillus, are rare in healthy individuals. Hospital-acquired diseases, including *P. aeruginosa*, account for approximately 10% of infections. Patients with burn wounds or cystic fibrosis are particularly susceptible to this type of infection. This opportunistic bacterial pathogen secretes several toxic proteins [91]. Among these, Exotoxin A is the most powerful and has certainly been the most studied.

Exotoxin A (PEA) is a highly toxic protein. Its intrinsic toxicity is due, once secreted, to its ability to translocate the catalytic domain inside mammalian cells to inhibit protein synthesis through chemical modification of elongation factor 2 (EF2) via ADP-ribosylation; PE thus acts as an adenosine diphosphate (ADP) ribosyltransferase [92,93].

**Figure 3:**
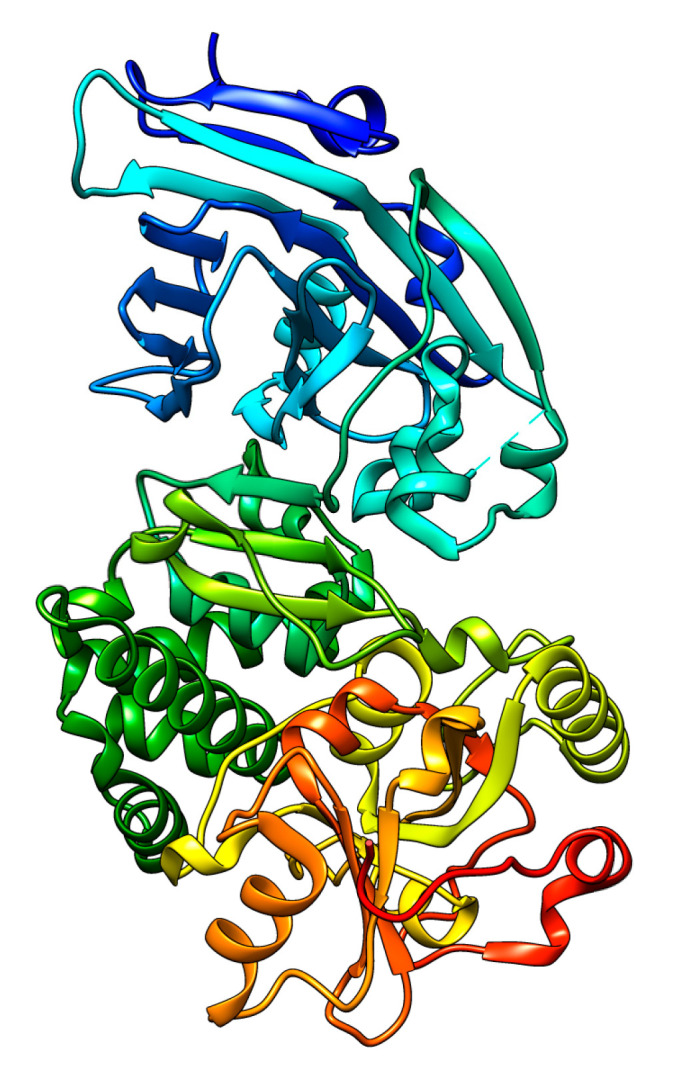
The crystal structure of Exotoxin A (PDB code: 1IKQ). PEA is formed via a receptor-binding (blue), translocation (green), and catalytic domain (red). The structure was created using the UCSF Chimera Software (1.17.3).

The N-terminal peptide of the 638-residue polypeptide PE toxin is eliminated during the process of bacterial cell secretion. Thus, the mature PEA toxin consists of a single 613 amino acid polypeptide chain with four disulfide bridges. The polypeptide chain comprises three domains (DI, DII, and DIII), each of which has a distinct function, as seen in Figure 3 [94].

The DI domain is responsible for binding to cell membranes through the interaction with low-density lipoprotein-receptor-related protein (LRP1) and related receptors [94]. The DII domain is involved in toxin translocation [93] following receptor-mediated endocytosis internalization. 

The DIII domain (405–613 amino acids) harbors the PE molecule catalytic center, has ADP ribosyltransferase activity, and is responsible for the inhibition of cellular protein synthesis. Through ADP ribosylation, the PE molecule transfers the ADP group from NAD+ to the cellular EF2 to form ADPR-EF-2, which inactivates the ribosomal elongation factor [94]. The blockade of protein synthesis results in cellular apoptosis. For several years, PEA toxin has been produced and used for therapeutic purposes, especially in the field of oncology as an immunotoxin (IT) component, chemically or genetically linked to monoclonal antibodies (mAb) or mAb fragments recognizing tumor-specific antigens [93].

#### PE Expression Hosts

- PE Bacterial expression systems

A lot of PE-based chimeras have been described in the literature, conjugating PE and its derivatives with various antibody formats, such as single-chain antibody (scFv), disulfide-stabilized antibody (dsFv), bispecific antibody, micro-antibody, and trivalent antibody, and have yielded promising results in both clinical and preclinical tests. This conjugation has led to the development of mono- or bivalent immunotoxins, demonstrating significant potential in the field [93,94,95,96,97]. 

**Figure 4:**
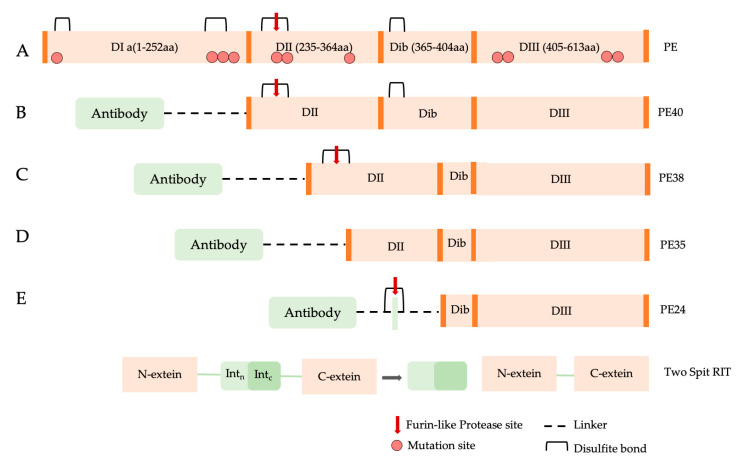
PE and derivative structure of the PE and derivative structure of PE- immunotoxins diagram (**A**–**E**). There are three domains in the full-length PE toxin. The size of the toxin decreased progressively as its structure was optimized, but domain III was maintained. Split intein was also used to create immunotoxins through an in vitro trans-splicing reaction. The figure was adapted from [97].

To construct recombinant PE immunotoxins, different optimization strategies are used based on the reduction in toxin size or in vitro trans-splicing through the intein split reaction [97] (Figure 4).

Amino acids 57, 246, 247, and 249 of the PE molecule can be changed to glutamic acid to limit the toxin’s binding capacity, removing its ability to target and bind cells by itself [98] (Figure 4A). In the PE40 derivative, the DI domain has been removed and substituted with antibody fragments, and several mutations (amino acids 276, 279, and 330) have been introduced to reduce toxicity (Figure 4B), while in the PE38 derivative (Figure 4C), amino acid residues 365–380 in the nonfunctional domain have been additionally removed, and the intramolecular disulfide bond has been broken to increase the IT tolerance in vivo experiments [99]. The PE35 derivative, amino acid residues in structural domain II (253-279aa), were deleted, and all disulfide connections in the molecule were broken, but the activity was maintained, and immunotoxin cell tolerance improved (Figure 4D) [98]. PE24 (Figure 4E) is a PE derivative widely used in immunotoxin construction because of the removal of domain DII, excluding the cleavage site of furin (FCS), decreased immunological recognition, and vascular leak syndrome (VLS) [100]. Split inteins were also used to create PE immunotoxins, such as scFvPE38 [101] and an anti-HER1/2 immunotoxin [102]. Enzymes known as inteins are able to splice out of other protein sequences in which they are embedded. This process involves splicing the two flanking polypeptides, or exteins, together using a peptide bond. Even after splitting spontaneously or artificially into N- and C-terminal fragments, split inteins can still combine to form a functional bipartite enzyme that can catalytically splice two separate extein polypeptides together.

Several PE-based recombinant toxins are currently in development for the treatment of cancers, but the main challenges to the successful clinical use of PE-RITs continue to be immunogenicity and non-specific toxicity [97], for which some strategies have been developed, such as, for example, the de-immunization via deletion and/or substitution of the immunogenic residues [103,104,105,106], modification with polyethylene glycol (PEG), and a combination of treatments to reduce B- and T-cell reactivity against the antigenic epitopes [97]. 

Two hosts are employed to generate PE-based toxins: the main one is *E. coli* [107], which guarantees efficient production with high yields and low cost on a large scale; the alternative is to use the native host, *Pseudomonas aeruginosa* [108].

By genetically engineering scFvs, comprising the heavy- and light-chain variable regions (VH and VL), with a truncated 38-kDa mutant form of PE, PE38KDEL, one of the first recombinant PE-based ITs was created in *E. coli* [109]. The resulting expressed protein only contains the translocation domain II and a catalytic subunit (domains Ib and III), as the cell-binding domain and portions of the translocation domains II and Ib are not necessary for the cytotoxicity of the toxin. The recombinant protein was expressed in inclusion bodies and as a soluble form using *E. coli*, specifically the BL21 (DE3) strain, as the expression host; in this case, the expression of the soluble form was more successful [109].

More recently, a new recombinant IT containing PE38KDEL with dual recognition towards the EGFR receptor, both as the wt and mutant EGFRvIII (D2C7-IT), has been obtained. In this work, D2C7-IT was expressed in *E. coli* BLR (λ DE3) under the control of the T7 promoter. D2C7-IT was extracted from inclusion bodies via anion exchange chromatography purification, followed by size exclusion and final endotoxin removal. The final yield was more than 30 mg/L [110].

In recent work, Zanjani and colleagues produced a nanoconjugate vaccine called Exotoxin A-PLGA against *Pseudomonas aeruginosa* infection. A standard strain of *P. aeruginosa*, called PAO1, was used to produce PE with standard protocols, resulting in 0.2% formaldehyde and 0.05 M L-lysine incubation at 37° for one week [108].

- PE Yeasts expression systems

In work from 2015, Della Cristina et al. engineered and expressed in *E. coli* and in *P. pastoris* several recombinant chimeras in which an scFV derived from a 4KB128 anti-CD22 murine IgG_1_ antibody was fused to PE40 and saporin, and it was shown that PE40-based IT was better expressed in the *E. coli* host compared to saporin-based IT that was better expressed in yeast after codon optimization [111]. Actually, in the literature, there are no more reported data concerning the use of yeasts as hosts for PE recombinant production.

- PE expression in Algal chloroplasts

Plant expression systems have been explored as alternative hosts for the production of recombinant immunotoxins (RITs). This involves utilizing them as hosts for generating inactive precursor proteins and sequestering them within chloroplasts to mitigate cellular toxicity.

**Figure 5:**
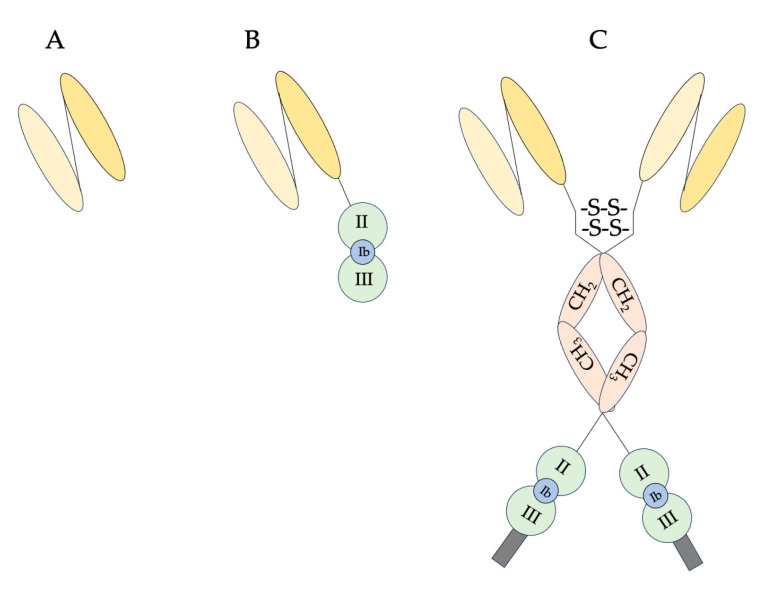
A single-chain antibody (**A**) can be fused to exotoxin A to produce two distinct immunotoxins: a monovalent immunotoxin (**B**) and a divalent immunotoxin (**C**), in which an Fc domain from a human IgG1 is inserted between the single-chain antibody and the exotoxin A. The figure has been adapted from [112].

It has indeed been demonstrated that the green alga *Chlamydomonas reinhardtii* chloroplast contains the molecular equipment required to fold and assemble complex eukaryotic proteins. However, chloroplasts can acquire eukaryotic toxins that would normally kill their eukaryotic hosts since their translational apparatus is similar to that of a prokaryote. An interesting work [112] described the production of a fusion protein containing the enzymatic domain of exotoxin A from *Pseudomonas aeruginosa* and an antibody domain targeting a B-cell surface epitope (CD22) in *C. reinhardtii* chloroplasts (Figure 5). Chloroplasts were transformed using genes expressing monovalent and divalent immunotoxins with an antibody-binding domain and a toxin derived from a eukaryotic source.

## 3. Plant Toxins

### 3.1. Saporin SO6

One of the most widely used type I ribotoxins (RIPs) for the development of therapeutics is saporin, which is obtained from the seeds of the *Saponaria officinalis* plant [113]. In the production of immunotoxins or chimeric toxins, saporin-6 (SO6) was chosen for toxin synthesis among the variety of saporins that *Saponaria officinalis* expresses because, when compared to other type I ribotoxins [114], it is stable and resistant to several modifications, such as denaturation and proteolysis [115], and for the maintenance of its enzymatic activity after conjugation procedures [116].

**Figure 6:**
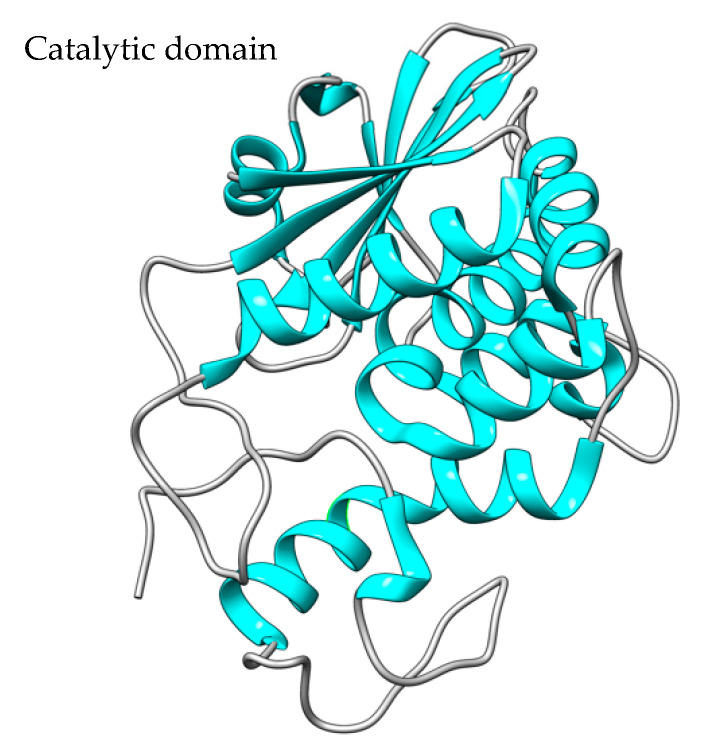
The crystal structure of saporin SO6 (PDB code: 1QI7). Saporin contains a single N-glycosidase catalytic A domain. The structure was created using the UCSF Chimera Software (1.17.3).

Saporin-S6 (Figure 6) has a full length of 253 amino acids. The sequence was identified in 1990, and lysine residues account for about 10% of the total amino acids, which allows the protein to have an exceptionally high pI (around 10) [117].

The saporin-S6 molecule contains no neutral sugars, notwithstanding the presence of glycosylation sites in the precursor pro-saporin C-terminal sequence, which is removed to produce the mature protein. Saporin and all plant RIPs have N-glycosidase activity (EC 3.2.2.22), which removes an adenine (A4324 in the 28S ribosomal rRNA) from the sarcin/ricin loop, a universally conserved GAGA-tetra loop [118]. This depurination process permanently deactivates the major ribosomal subunit by preventing the recognition and binding of the elongation factor EF-2 and influencing both EF1 and EF2 GTPase activity. This prevents tRNA translocation from the A to P sites, irreversibly stopping protein synthesis [119,120,121]. Saporin-S6 has demonstrated harmful effects in both in vivo studies using animal models and in vitro experiments involving various cell lines.

Saporin-S6 has an LD_50_ of 4.0 mg of RIP/Kg of body weight in mice. It can activate apoptosis (both caspase-dependent and -independent apoptosis), oxidative stress, autophagy, necroptosis, and protein synthesis inhibition once it enters the cytosol, ER, or nucleus. When full-length antibodies are used, the presence of the antibody itself can trigger cell death through apoptosis, complement-dependent cytotoxicity (CDC), or antibody-dependent cellular cytotoxicity (ADCC) in saporin-based ITs [122].

Type 1 RIPs have low inherent toxicity and lack a cellular entrance mechanism. Type 1 RIPs become highly toxic when linked to a cell recognition and entrance element, and they have shown significant action against hematological and solid malignancies [123,124,125,126,127,128,129,130,131,132]. Saporin S6 constructs have been shown to go straight from endosomes to the cytoplasm, whereas ricin conjugates have been shown to move to the Golgi from endosomes, then to the endoplasmic reticulum, and finally to the cytosol [121]. 

In the literature, there are a lot of works that describe the design and construction of RIP-based immunotoxins for treating cancer, HIV, and other infectious diseases [122,133,134,135,136], including saporin. Classical immunotoxins were first produced via chemical cross-linking [116], but the need for more homogeneous and reproducible products has led to the study of recombinant forms.

The effective production and appropriate folding in a host cell is an important feature for the development of therapeutic drugs for saporin chimeras (as well as that of plant RIPs chimeric toxins). To express plant protein toxins, particularly saporin or Type I RIPs recombinant fusion chimeras, a eukaryotic expression system would thus be desirable to drive their expression as secretory proteins so that correct folding with the cellular quality control system would be easiest in the endoplasmic reticulum (ER) microenvironment. These expression systems, however, face important problems mainly due to ER stress and the intrinsic toxicities of plant toxins to eukaryotic ribosomes.

#### Saporin Expression Hosts

- Saporin Bacterial expression systems

While *Ricinus communis* [137,138], *Mirabilis jalapa* [139], *Phytolacca americana* [140], *Dianthus* leaves [141], *Trichosanthes kirilowii* [142], *Maize* [143], and *Gelonium multiflorum* [144] RIPs have been cloned and expressed in *E. coli*, saporin expression in bacteria is more difficult because of the direct action on the host ribosomes that may cause autointoxication; in contrast to type II RIPs, type I RIPs, and particularly saporin, are active against both eukaryotic and prokaryotic ribosomal RNA [145].

Additionally, although several *E. coli* vector designs for the expression of saporins were tried, they all showed severe toxicity in *E. coli* non-expression strains during plasmid maintenance and strain selection. Toxin expression might be finely controlled using the *E. coli* strain BL21 (DE3) pLysS, but it is not possible to prevent endotoxin contamination and the expression of toxins in insoluble form [115].

In a 1993 study, the group of Barthelemy described the amplification of genomic DNA from the leaves of *S. officinalis* and the expression in *E. coli* of a PCR-amplified genomic clone of saporin S3 and S6 [146]. The saporin coding sequence was inserted into the periplasmic secretion vector PIN-IIIompA2, resulting in a gene fusion between the mature saporin coding sequence and the plasmid ompA leader peptide segment to direct membrane translocation in *E. coli* and assist proper protein folding of the expressed protein. Most of the saporin expressed showed similar protein synthesis inhibition activity to the native saporin but remained inside the cells, and approximately only 10% was found in the periplasm [146]. 

Fabbrini et al. expressed for the first time in *E. coli* five isoforms of saporin: SAP1, SAP3, SAP4, SAP6, and SAP-C [147]. For the expression of recombinant proteins, the authors used the BL21 (DE3) pLysS strain and the pET11d plasmid to engineer the saporin genes. The saporin-coding leaf cDNA [148] has been modified to include a stop codon before the transcribed C-terminal propeptide [149]. After that, the resulting construct, pET-11d-SAP-C, was completely sequenced to ensure that no modifications were introduced during the amplification stage. The protein expression in the soluble fraction has been possible with the use of a tightly regulated host/vector inducible system; there was no evidence of leaky expression of saporin genes prior to T7 RNA polymerase activation, and yields of soluble recombinant seed-type saporin isoforms were between 1 and 3 mg/liter of culture [147].

The substitution of Glu 176 with Lys and the deletion of 19 amino acids at the C-terminal of the saporin sequence have been used to express two saporin mutants in *E. coli* to reduce its toxicity to bacterial cells and affect its enzymatic activity on polynucleotide substrates. Bacterial cultures carrying wild-type recombinant saporin (pET-Sap) slowed to a halt, whereas cultures having pET-E176K and pET-K234stop mutant variants grew normally and at the same rate as control cultures, but the majority of recombinant saporin mutants appeared as inclusion bodies [150].

Günhan et al., in 2008, described the expression and purification of saporin derivatives in *E. coli* BL21(DE3)pLysS cells, a strain used for the expression of toxic proteins because it encodes T7 lysozyme, which suppresses the basal expression of toxic target proteins prior to induction. The authors introduced a mutation at the C-terminus (Ser255Cys) of the gene to permit the conjugation reaction of a heterobifunctional crosslinking agent to antibodies, cytokines, peptides, and the construction of conjugates. The Cys255-Sap was efficiently recovered from the soluble fraction (12.5 mg/120 mg total proteins), reaching 95% purity and a yield of 2.7 mg/L after the size-exclusion chromatography step [151]. As previously indicated, Sap S6 shows great resistance to chemical modification, denaturation, and proteolysis, so it is a very good candidate for the construction of conjugates for therapeutic purposes.

Giansanti et al. have developed, generated, and characterized a RIP saporin (SapVSAV) engineered form with an additional C-terminal sequence (SEVSAV) that is recognized using the PDZ2 domain of mouse protein tyrosine phosphatase [152]. The co-expression of PDZ2 and the mutated saporin gene boosts toxin production in *E. coli* BL21 strain cells and gains an expression level similar to what is found in the PlysS-protected system. The authors accomplished co-expression using pET28 and pET11 vectors but also achieved sustained co-expression using a bi-cistronic pETDuet plasmid containing both the two gene sequences. The induction of SapVSAV expression did not affect bacterial growth, maybe because it was produced in an inactive form stabilized through the PDZ2 interaction, but when SapVSAV was evaluated in cellular systems (Daudi or U937 cells), the authors found that it had cytotoxic activity comparable to wt saporin, implying a putative activation mechanism induced in mammalian cells. 

The same group in 2015 produced recombinant chimeric toxins composed of the PDZ-hCASK (serine kinase calcium/calmodulin-dependent of the MAGUK family binding to CD98) sequence coupled to saporin S6 as monovalent (hCASK-SAP) or bivalent (hCASK(2SAP) toxins and tested their toxicity towards human glioblastoma cell lines (GL15 and U87) [153]. The synthetic genes hCASK and SAP S6 were fused, cloned, and expressed in Rosetta Gami^TM^ B pLysS(DE3) *E. coli* strains, which combine enhanced disulfide bond formation with increased expression of eukaryotic proteins containing rare *E. coli* codons. The expression yields of both chimeras were not as efficient as those of the hCASK domain alone, but they showed higher toxicity on two glioblastoma cell lines (U87 and GL15), especially in the presence of saponin as a “coadiuvant” for the endo/lysosomal compartment escape of the toxins [154]. 

Recently, a new saporin conjugate production has been described in the literature. The saporin gene has been genetically fused to the ACDCRGDCFCG peptide (RGD-4C), an αv-integrin ligand, and expressed in *E. coli* with a histidine tag at the C-terminus to promote endosomal escape. This conjugate was tested in in vivo studies in different orthotopic mouse models of bladder cancer and was able to reduce tumor growth and significantly prolong animal survival. The RGD-SAP was easily recovered from the bacterial soluble fraction as a monomer with no need for renaturation steps [155].

- Saporin Yeasts expression systems

Although the *E. coli* strain BL21 (DE3) pLysS could be used to regulate RIP expression more tightly, their high toxicity and the processing of inclusion bodies remain problematic, and recombinant saporin-IT production has been extensively described in yeasts [156]. Yeasts exhibit diminished nutritional needs in comparison to insect and mammalian cell lines and furthermore combine the simplicity of a unicellular organism with the capacity to carry out most post-translational modifications needed for a physiologically active recombinant protein. *P. pastoris* is an obligate aerobic yeast that can obtain carbon from methanol. This last feature enabled the construction of an expression system exploiting the methanol-inducible AOX1 promoter. When compared to *S. cerevisiae*, *P. pastoris* is recognized for producing a higher number of recombinants because it is Crabtree-negative, avoiding loss of carbon through the production of ethanol under respiratory circumstances, resulting in more biomass creation and, subsequently, more recombinant protein [157]. 

In the aim of resolving some important issues of the first-generation RIP-based ITs and imposing several advantages, such as a defined toxin-ligand interface and the possibility to genetically edit mutations in the recombinant toxin to boost potency and reduce nonspecific toxicity [158,159], recombinant ITs were created using single-chain variable fragments (scFvs) as the carrier moiety, later replaced by disulfide-stabilized Fvs (dsFvs) [160,161].

To achieve optimal expression of saporin and relative fusion chimeras in *P. pastoris*, an important factor that must be considered is the use of optimization of the coding sequence on the basis of yeast codon usage. Saporin S6 was initially expressed by the Fabbrini group, both as a standalone toxin and as part of a fusion immunotoxin in *P. pastoris* [162], and then the same group produced a chimera consisting of the ATF of human uPA fused to the saporin S3 isoform that specifically kills uPAR (urokinase plasminogen activator receptor) over-expressing cancer cells [163].

In an interesting paper from 2015, Della Cristina et al. [111] showed the construction and recombinant production of two various constructs containing the identical recombinant anti-CD22 single-chain variable fragment (scFv) fused to two different toxin domains: PE40 or saporin. Both immunotoxins were expressed in *E. coli* and in *P. pastoris*. On a small scale, the PE40 chimeras expressed in *E. coli* accumulated in inclusion bodies, and no measurable recombinant immunotoxin (rIT) could be retrieved in a soluble form, either within the cytoplasmic or periplasmic spaces. The renaturation of inclusion bodies in a larger culture volume showed a recovery of about 3 mg/L of the immunotoxin, corresponding to 80% of the total expressed protein [114]. In the case of the saporin domain containing rIT, the authors found a lower level of immunotoxin production than that observed for the rIT containing PE40 following IPTG induction in *E. coli*, not due to host auto-intoxication effects. Even though this saporin chimera could be recovered from inclusion bodies at a concentration of 4 mg/L, most (>90%) of the toxin was lost during the renaturation process as a result of aggregation and precipitation events. Having obtained low- and non-functional quantities of this saporin-based IT in bacteria, the authors decided to design some constructs suitable for *P. pastoris* expression, fusing the sequences coding for the anti-CD22 V_H_ and V_L_ domains to a saporin yeast-optimized for codon usage, either engineered to have an N- or C-terminal His-tag. Codon optimization has previously been demonstrated to significantly reduce the toxicity challenges related to saporin expression in *P. pastoris*, as well as to be required for producing clones that express high levels of active recombinant saporin [156]. In particular, it has been shown that misfolding may occur when saporin is fused to an “unfavorable” domain, such as the scFv, resulting in increased host toxicity and lowering expression levels. Codon-usage optimization could counteract such an effect, improving the expression yield in *Pichia* even in the presence of the scFv domain. The estimated secretion yields of secreting colonies were about 1–2 mg/L, and the proteins have full cytotoxic activity, suggesting that *P. pastoris* is a better host for saporin-based rITs than *E. coli.* Codon usage optimization is a strategy also used for the design and production of the ATF-SAP chimera in the fermentation process [164]. Between the 14 (fully optimized ATF-SAP) and 15 (only optimized SAP) *GS115* clones producing ATF-SAP, the expression levels vary from 1 to 5 mg/L for the partially optimized chimera and from 3 to 7 mg/L for the fully optimized chimera. A feeding strategy based on the fed-batch slow addition of methanol and on the oxygen transfer rate increase allows the yeast cells to adapt smoothly to methanol after induction, so the concentration of the secreted recombinant ATF-SAP reached approximately 6 mg/L, demonstrating the tolerance of this strain towards SAP. Further advancements would be achievable by improving pre-induction biomass formation, as is possible in industrial-scale bioreactors.

- Saporin expression in Tobacco protoplasts

The use of plant tobacco protoplasts has been investigated as an alternative approach for producing recombinant plant RIPs. However, it was discovered that the saporin precursor is extremely harmful to this expression system [165]. Interestingly, tobacco protoplasts were able to express the native preproricin construct, and normal processing, glycosylation, and targeting of the vacuole took place with no harmful effects found [166]. In contrast, when an orphan secretory ricin toxic A chain (RTA) polypeptide was expressed, RTA was retro-translocated to the cytosol, and protein synthesis was thereafter inhibited [166]. This further suggests that RIP toxicity may have negative effects on the tobacco protoplast expression system. The purified toxin showed protein translation inhibitory activity similar to the native one only when the expressed precursor contained the C-terminal propeptide [167], suggesting a fundamental role of the latter in the segregation of this RIP. It was demonstrated in other RIPs, pre-pro-trichosanthin, that proper processing during the expression of transgenic tobacco plants was dependent on the presence of the two peptides:

- Gene therapy with saporin gene

Suicide saporin-gene therapy has been studied on various cell lines (B16, Hela, U87, and MDA-MB-435), using the toxin gene carried in plasmids and expressed under the control of cytomegalovirus (CMV) or simian virus 40 (SV40) promoters [168]. B16 melanoma cells transfected with the pCI-SAP plasmid showed a dramatic reduction in growth of about 70% with respect to controls, and also in B16 melanoma-bearing mice, direct intra-tumoral injection of pCI-SAP resulted in a significant reduction in tumor growth [169]. Co-transfection treatment of HeLa, U87, 9L, and MDA-MB-435 cells with two distinct mammalian gWIZ plasmids, pGEL (gWIZgelonin) and pSAP (gWIZ-saporin), demonstrated strong cytotoxicity on all the cell lines tested using a DNA concentration of only 2 µg/mL. However, due to the non-selective DNA delivery system, the toxin expression caused cytotoxicity in both malignant and non-cancer cells [170]. Some examples of selective delivery systems have been described more recently in the literature. The antitumoral activity of a plasmid harboring the saporin gene bound to lipid-protamine DNA nanoparticles coated with a peptide directed against human urokinase (U11) was established in mouse models [171]. With a similar strategy, di Leandro L. et al. tested an aptamer-mediated (AS1411 targeting surface nucleolin) saporin gene delivery system against U87 glioblastoma cells. A 50% reduction in U87 viability was achieved using an average saporin DNA concentration of 24–30 μg/mL, demonstrating selective cytotoxicity on glioblastoma U87 cells without toxic effect in 3T3 control cells [172].

### 3.2. Ricin A Chain (RTA)

Ricin, a type II RIP mainly purified from the seeds of *Ricinus communis*, is made up of two different polypeptides (the A and B chains) held together by a disulfide bond. The B chain is a galactose-binding lectin that ricin uses to bind to cell membranes, whereas the A chain is a N-glycosidase (EC 3.2.2.22) that, once delivered to the cytoplasm via retrograde transport through the Golgi and ER, kills the cell by catalytically inactivating the 60 S ribosomal subunits [173,174]. 

Numerous scientists have attempted to use ricin’s high cytotoxicity to kill cancerous cells for medical needs. Despite having extremely effective cell-killing capabilities, ricin is not selective for cell targets. The prospect of coupling ricin to carriers specialized for targets on undesirable cells has been extensively investigated in an effort to improve selectivity. In a cell culture test system, the ricin A chain (RTA) shows less than 0.01% toxicity of the naturally occurring protein, has no effect on both non-infected and TMV-infected tobacco protoplasts, and is unable to enter the cell without the help of the B chain [175]. But due to its great potency in blocking protein synthesis, the A chain (RTA) is extensively used to build cytotoxic conjugates effective against tumor cells as immunotoxins and fusion proteins [176,177,178].

There are many studies in which immunotoxins and RTA-based chimeras were proposed and designed against various cancer conditions, in particular against refractory hematologic malignancies [179,180]. A recombinant anti-IL-2R IT carrying deglycosylated dgRTA has obtained approval from the US Food and Drug Administration for the treatment in adults of cutaneous T-cell lymphoma [180], and several others are in clinical trials (Combotox, 3A1-dgRTA, IgG-HD37-dgA, Xomozyme-791, RTF-5-dgA, 260F9-rA, H65-RTA) [181,182,183]. For a more exhaustive view of the clinical evaluations of ricin-based immunotoxins, see also the review of de Virgilio M. et al. [184].

Native RTA must be effectively separated from the ricin B chain (RB), which binds to structures on the cell surface that contain galactose and facilitates the entry of RA into the cytoplasm, in order to be purified from the entire toxin [159]. One issue with employing A chains produced from fully native ricin is that laborious and extensive methods are required to remove all the contaminating B chains, procedures that are required to avoid non-specific toxicity [185]. A second issue is that the ricin A chain is N-glycosylated and must be deglycosylated to prevent immunotoxins from being cleared quickly in vivo by liver cells that carry mannose receptors. To prevent even minute levels of contaminating toxin or RB that can hide the hybrid toxin’s target selectivity and raise overall toxicity, preparations of RA and its conjugates must be closely regulated. Additionally, dealing with a lot of ricin and castor beans puts your health at risk [185].

To circumvent these issues and to more affordably obtain large quantities of RTA for therapeutic development, genetic methods are used to express RTA or deglycosylated RTA (dgRTA) genes in *E. coli*, as reported below.

To date, recombinant RTA and dgRTA have been used to obtain immunoconjugates in which the antibody is chemically coupled to the toxin [178]. 

Moreover, in the literature, few examples of RTA immunotoxins produced by genetic fusions in *E. coli* are reported [186,187]; instead, there are several examples of RTA chimeras that have been successfully expressed and tested for anticancer activities. 

#### RTA Expression Hosts

- RTA Bacterial expression systems

**Figure 7:**
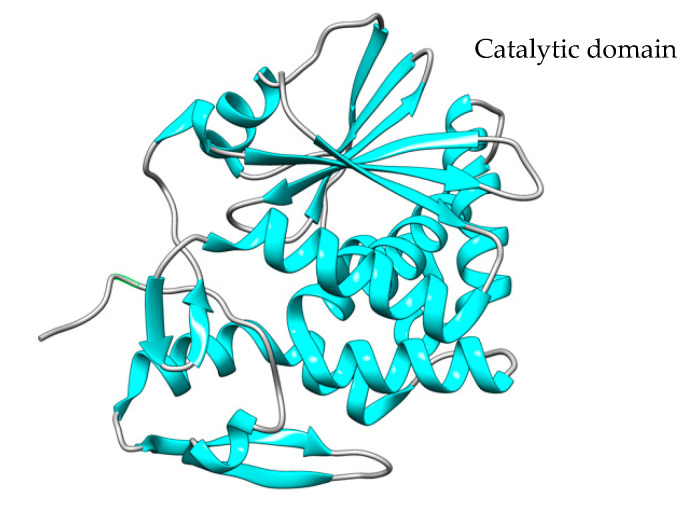
The crystal structure of the A chain (PDB code: 4Q2V). The catalytic domain is represented in blue. The structure was created using the UCSF Chimera Software (1.17.3).

The structure of the isolated A chain expressed in *E. coli* is reported in Figure 7 [138,188].

The ricin A chain was expressed for the first time in *E. coli* in 1987 [189]. In the same year, it was also described as the construction of the first recombinant immunotoxin based on RTA: in a nude mouse model of human ovarian cancer, the growth of OVCAR-3 tumors was shown to be inhibited by an immunotoxin made up of an antibody to the human transferrin receptor (454A12) and the ricin A chain (RTA) [190]. Similarly, one of the first examples of recombinant chimeras containing RTA was reported in a 1994 study in which the expression in *E. coli* of a chimeric toxin prepared via genetic fusion of RTA and DTA produced a partially soluble toxin; in fact, only 20% of RTA-DTA was found in the lysate supernatant, while most were insoluble. The chimera was then tested for cytotoxic activity against human ovarian cancer cells, OVCA433 [191]. To enhance the cytotoxicity of recombinant RTA, several methods have been optimized, such as the addition of a TGN retention signal YQRL to the C-terminus of RTA [192] or an endoplasmic reticulum retention sequence KDEL [193,194]. Furthermore, in cases of some RTA-based chimeric toxins, a protease-sensitive cleavage site has been inserted between the RTA sequence and the other protein sequences [195,196]. 

Recombinant immunotoxins are chimeric proteins made of a single-chain antibody fragment (scFv) and a shortened, binding-deficient, catalytically active toxin. These fusion gene products are more readily modifiable, more readily produced, and more homogeneous than chemical conjugates [197]. Single-chain immunotoxins produced by bacteria vary greatly in terms of stability, and some have shown a marked propensity for aggregation [198]. To increase solubility, different strategies can be used, some involving modification of the target (as the use of tRNA complementation plasmids and stabilization of mRNA), others involving modification of the growth conditions (pH, temperature, media, addition of molecular chaperones, etc.) [199].

To develop novel anticancer Ribosome Inactivating Toxin-Affibody Fusions (RTA RITs), Park et al. genetically combined an affibody coding sequence directed either to HER2 (ZHER2:342; HER2Afb) or EGFR (ZEGFR:1907; EGFRAfb) with the RTA N-terminus (residues 36–302). This fusion was engineered to carry the KDEL signal peptide and expressed in *E. coli* as HER2Afb-RTA-KDEL and EGFRAfb-RTA-KDEL [187]. To generate both HER2Afb-RTA and EGFRAfb-RTA, the RTA gene was cloned into a pETDuet-1 vector carrying either the HER2Afb or EGFRAfb gene, adding a C-terminal histidine tag for easier purification. Each of the recombinant protein-encoding plasmids was separately introduced into the BL21 (DE3) strain of *E. coli,* and the recombinant proteins were then overexpressed via induction with IPTG at 18 °C overnight. In this way, the proteins were recovered from the soluble fraction of the cell extract.

**Figure 8:**
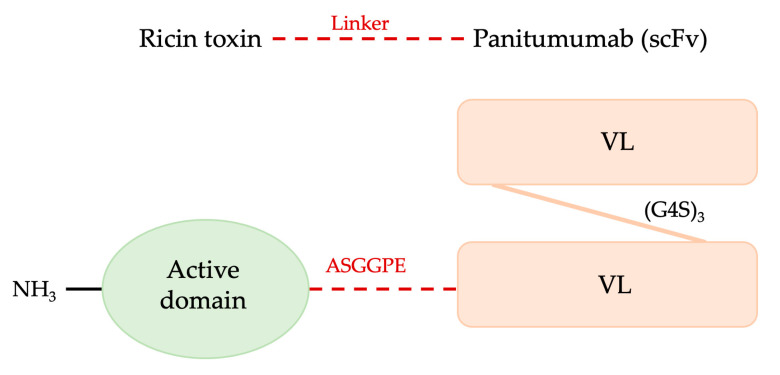
Schematic representation of the EGFR-specific recombinant ricin-panitumumab (scFv) immunotoxin. The figure has been adapted from [186].

A new immunotoxin (Figure 8) has been newly developed by combining the single-chain variable fragment (scFv) obtained from panitumumab. This scFv comprises the VH and VL regions and is fused with the catalytic domain of ricin (RTA). The new construct sequence was optimized for expression in *E. coli* strain BL21 (DE3) and inserted into the pET32a (+) expression vector. The fusion protein has been refolded through dialysis from inclusion bodies and purified at a concentration of about 0.18 mg/mL. This immunotoxin is designed against the Epidermal Growth Factor Receptor (EGFR)-induced cytotoxicity and apoptosis in HCT-116 and MDA-MB-468 cells [186].

In an effort to target infected cells and recognize viral components specifically, fusion and hybrid proteins of RTA and PAPs have also been produced [200,201]. A fusion protein between ricin A chain (RTA) and Pokeweed antiviral protein (PAP) isoform S1 (from the seeds of *Phytolacca americana*) was produced in an *E. coli* expression system and assayed for its anti-HBV inhibitory function and cytotoxic effect in the chronically infected cell line AD38 [202]. The chimeric toxin was purified from inclusion bodies with a yield of about 200 mg/L of culture with 90% purity. To improve its production in *E. coli*, the authors also produced an RTA mutant-Pokeweed antiviral protein isoform 1 from the leaves of *Phytolacca americana* (RTAM-PAP1). Two specific point mutations were incorporated into the RTA moiety as well as in the flexible linker to substitute the cysteine (Cys) residues with alanine residues. This modification was implemented to prevent the unwanted formation of disulfide bonds at positions 171 and 259 entirely (C171A and C259A). Additionally, to reduce any potential impact on its structure and function, a 6-His tag was added at the N-terminal of the RTAM-PAP1 protein. These modifications really made a difference in solubility and activity: the fusion proteins RTAM-PAP1 were produced exclusively with great solubility (a few were found in inclusion bodies), and using a three-step purification approach, soluble proteins with >90% homogeneity were obtained. Nevertheless, from 1 L of culture, 0.1 mg of protein with >95% purity and 0.22 mg of protein with >90% purity were recovered, but at the same time, the anti-HBV bioactivity of RTAM-PAP1 was increased with respect to RTA-PAP1 protein. 

As previously discussed, the potential toxicity of RIPs towards the host cells may prevent or decrease recombinant protein production and their use as such or included in chimeric proteins or in ITs, but since RTA does not exhibit toxicity towards prokaryotic ribosomes, it could be easily produced in *E. coli* with high yields and with minimal difficulty. Consideration should be given to how this catalytic domain is released into the cytoplasm. Chimeric toxins containing retinoic acid (RA) necessitate intracellular proteolytic cleavage for the liberation of the RA component, enabling them to exhibit cytotoxic effects on target cells. If the recombinant chimeric toxin undergoes extracellular cleavage, it fails to selectively target cells in a specific manner [203].

In order to enable appropriate and distinct folding of both domains of the chimeric toxin, this issue may be addressed by inserting a flexible peptide linker between the targeting domain and the toxic moiety. The 218 linker GSTSGSGKPGSGEGSTKG and the G4S peptide linker are used to improve the chimeric proteins’ resistance to intracellular proteases and even further decrease scFv (antibody single chain variable fragment) aggregation when expressed in bacterial systems [111,204]. 

### 3.3. Other Plant Toxins

It is worth noting that besides the ricin A chain and saporin, widely studied RIPs, a couple of examples should be mentioned for the recombinant expression of toxins from plants.

#### 3.3.1. Bouganin

In the last few years, the single-chain ribosome-inactivating protein bouganin (from *Bougainvillea spectabilis* Willd.) has attracted renewed interest due to the possibility of engineering its amino acid sequence to decrease the immunogenic properties of these toxins, thus increasing its attractivity as a component of immunotoxins [205]. Bouganin is a type 1 ribosome-inactivating protein purified from the leaf extracts of *Bougainvillea spectabilis* Willd [2] as a 26 kDa single-chain protein. Bouganin exhibits characteristics typical of type 1 ribosome-inactivating proteins (RIPs), such as N-glycosylase activity and antiviral properties; it blocks protein synthesis in a cell-free system (with an IC_50_ of 10 ng/mL), but its efficacy on whole cells is comparatively lower than other type 1 RIPs. It was noted that the concentrations of bouganin needed to inhibit protein synthesis in human cell lines were significantly higher than those required in a rabbit reticulocyte lysate assay and did not exhibit toxicity in mice at the highest tested dose of 32 mg/kg [206]. Moreover, bouganin possesses distinctive characteristics, including a higher activity on DNA compared to ribosomal RNA, low systemic toxicity, and immunological properties that differ significantly from other RIPs. The limited non-specific toxicity of this protein to animals, when compared to all other identified and characterized toxins, enhances the therapeutic possibilities associated with bouganin; in particular, it represents a highly appealing tool for incorporation into immunotoxins and fusion chimeras [205].

- Bacterial expression systems

**Figure 9:**
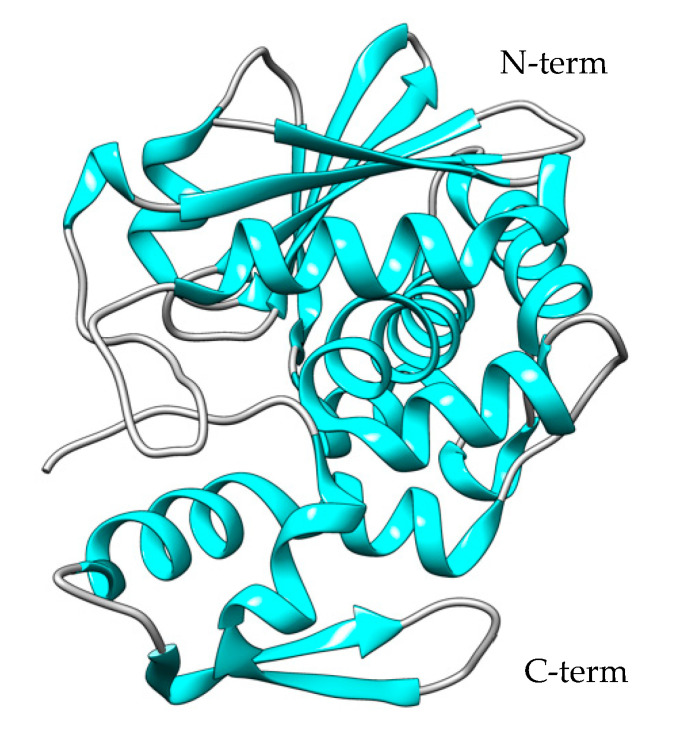
The crystal structure of bouganin (PDB code: 3CTK). The N-terminal domain consists of a mixed β-sheet of seven filaments (β1–β9). The C-terminal domain consists mainly of eight α-helices. The structure was created using the UCSF Chimera Software (1.17.3).

The first report of recombinant bouganin expression in *E. coli* was described in 2002 by den Hartog and co-workers [207]. The authors synthesized bouganin cDNA from total RNA isolated from the leaves of *B. spectabilis* Willd, and the full-length bouganin gene was directly amplified with PCR. The PCR product was cloned into a PG212 plasmid containing the pElB leader peptide for periplasmic expression, and the *E. coli* strain BL21-CodonPlus(DE3) was used as the host. Despite the presence of the pELB leader peptide, the majority of the recombinant protein was found in inclusion bodies, and only a small portion present in the periplasmic extract was purified through affinity chromatography. The most interesting aspect of this work was that the activity of recombinant bouganin was comparable to that of the native protein in living cells. This suggested that the recombinant production of bouganin does not alter its inherent difficulty in binding to cells, as evidenced by the high IC_50_ values shown in living cells compared to the cell-free assay. Bouganin’s 3D structure was solved with X-ray crystallography (Figure 9) and revealed a conserved structure typical of this class of toxins [208]. Interestingly, bouganin possesses two cysteine groups that can be employed to create a disulfide bond directly with an activated antibody thiol group through a disulfide exchange reaction. This method simplifies the generation of immunotoxin molecules with bouganin, suitable for use in novel therapeutic strategies.

On the basis of knowledge of the structural features of bouganin, it was possible to change some specific amino acid residues to impact the immunological properties of the toxin without affecting its catalytic activity [209]. Cizeau et al. mutated the bouganin gene to remove the T-cell epitopes to create a T-cell epitope-depleted variant of bouganin, named de-bouganin. By genetically combining de-bouganin with an anti-epithelial cell adhesion molecule (EpCAM) Fab moiety, the VB6-845 immunotoxin was obtained.

To test the most effective antibody–de-bouganin orientation, multiple versions of dicistronic expression units were created, expressed, and evaluated for potency. In all instances, the dicistronic unit was inserted into the pING3302 vector, regulated with the arabinose-inducible araBAD promoter, and introduced into the E104 *E. coli* strain. Upon induction, the PelB leader sequence facilitated the secretion of the Fab–de-bouganin fusion protein into the periplasmic space. The conjugate biological activity was tested against cell lines expressing EpCAM, CAL-27, and OVCAR-3; the immunotoxin displayed higher potency compared to numerous widely used chemotherapeutic agents. In vivo, effectiveness was validated through an EpCAM-positive human tumor xenograft model in severe combined immunodeficiency (SCID) mice, with the majority of treated mice remaining tumor-free [209].

Some other recombinant de-bouganin-based ITs have been designed and expressed in *E. coli*. The immunotoxin Trastuzumab (anti-HER2 mAb)–bouganin was used as a complement to the treatment of mammary gland ductal carcinoma cells resistant to treatment with maytansinoids [210]. An anti-HER2 C6.5 diabody–de-bouganin/de-bouganin–C6.5 diabody fusion protein was expressed and purified from *E. coli* with a yield of about 0.15–0.5 mg/L and then characterized for their activity on HCC1419 and BT-474 cells, where the fusion-carrying bouganin at the N-terminus proved to be more efficient in killing cells [211]. Recently, a phase I trial has been initiated using an antiEPCAM/de-bouganin fusion for the treatment of epithelial tumors [212]. The absence of immune reactivity to bouganin in patients underscores the effectiveness of the T cell epitope-depletion strategy in mitigating the immune response and confirms the feasibility of utilizing bouganin in therapeutic strategies.

#### 3.3.2. Pulchellin

Pulchellin is a ricin-like lectin (type II RIP) obtained from *Abrus pulchellus*. Pulchellin consists of a RIP A chain and a B chain linked by a disulfide bond [213]. The B chain, serving as a non-toxic carbohydrate-binding component, shows a crucial role in facilitating the endocytosis of the A chain and could be utilized as a mechanism for drug delivery. The catalytic residues within the pulchellin A chain are positioned identically to those found in the Ricin and Abrin A chains. Pulchellin exists in four isoforms, with isoform II being the most potent, exhibiting an LD_50_ toxicity of 15 µg/kg in mice [214].

The DNA fragment that encodes pulchellin A chain was cloned and introduced into the pGEX-5X plasmid for the expression of recombinant pulchellin A chain (rPAC) in *Escherichia coli* [213], with a good final yield after purification of about 3 mg/L.

The recombinant pulchellin A chain (rPAC) contains a single free cysteine situated in the C-terminal region, allowing it to readily engage in a disulfide-exchange reaction with an activated antibody thiol group. This characteristic facilitates the easy production of rPAC in a heterologous system. Consequently, rPAC holds promise for the development of immunoconjugates with significant potential as chemotherapeutic agents. The first example of using pulchellin in targeted therapy is described by the work of Sadraeian et al., in which the authors showed the expression and purification of immunotoxins made by chemically linking the toxin to antibodies directed against gp120 and gp41 expressed on the surface of HIV-envelope-producing cells [215]. The recombinant toxins were produced in *E. coli* Rosetta (DE3) and conjugated to HIV MAbs 924 and 7B2 with the single-free cysteine on the A chain toxin. The recombinant pulchellin was internalized by these cells, suggesting its possible use as a therapeutic agent against HIV-infected cells.

## 4. Animal Toxins

### Melittin

The development of new drugs derived from animal toxins represents the origin of an enormous series of therapies for clinical use. Spiders, scorpions, insects, and some marine animals produce toxins. Given their high and complex content of peptides, animal toxins are of increasing interest in relation to their potential therapeutic use. 

Several toxins from Conus (cone snails), arthropods (spiders, scorpions, centipedes, bees, etc.), vertebrates (snakes, lizards, etc.), and cnidarians (jellyfishes, sea anemones, etc.) are produced by recombinant techniques in bacteria and yeasts and have an important biotechnological potential as therapeutics, bioinsecticides, anti-cancer drugs, and anti-human pathogens [216,217,218].

To date, one of the animal toxins most efficiently produced by recombinant techniques is melittin from honeybee venom (BV). Melittin is a rare example of an animal toxin expressed recombinantly in various hosts and used not only because of its direct effects on cells but also as a part of conjugates and immunoconjugates, chimeras, and fusion proteins, and it is also used as a vehicle for gene therapy (Table 2). 

**Figure 10:**
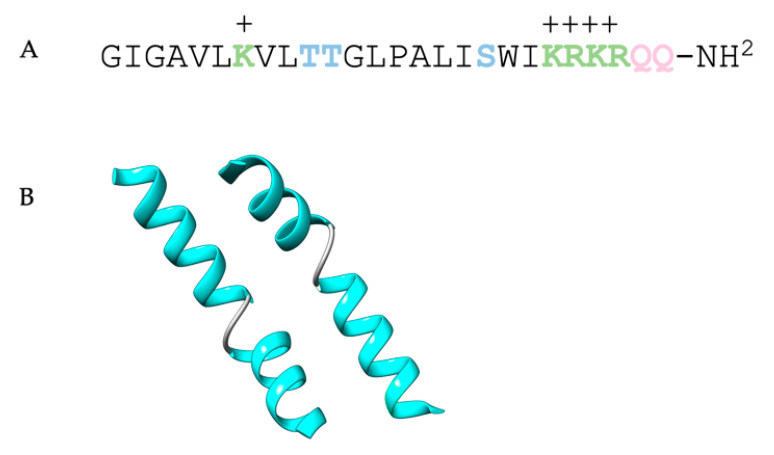
(**A**) Sequence and (**B**) crystal structure of melittin (PDB code: 2MLT). The structure was created using the UCSF Chimera Software (1.17.3).

Melittin is a small (26 amino acids) basic peptide originally purified from honeybee (*Apis mellifera*) venom. In this peptide, the amino-terminal region is primarily hydrophobic, while the carboxy-terminal region is mostly hydrophilic due to the presence of a segment with positively charged amino acids (see Figure 10).

Melittin binds to cell membranes via interaction with the lipid bilayer by folding into an amphipathic α-helical secondary structure and decreasing the permeability of the membrane itself [236], probably disturbing the segregation of polar and non-polar moieties across the bilayer, even though the exact process is still unclear [237,238] and may even vary in some cases [239]. Melittin may form stable transmembrane helical bundles [236] to generate holes sometimes while promoting temporary membrane permeabilization in synthetic bilayers under most circumstances because its equilibrium orientation is parallel to the membrane surface [240]. Melittin shows several mechanisms of action in different cell types, including anti-inflammatory, anti-arthritic, antiviral, and pain-relieving activities [241]. Additionally, it causes apoptosis, growth inhibition, and cell cycle arrest in certain tumor cells. It has undergone in vivo and in vitro testing with promising results for its prospective use as a treatment for cancers of the breast, ovary, prostate, and hepatocellular carcinoma [242,243,244]. Although melittin has the potential to be used as a cancer chemotherapeutic drug for a long time, its quick blood breakdown and non-specific cellular lytic action represent substantial difficulties [245]. Melittin has a strong toxic effect when administered intravenously, such as hemolysis [246], which prevents it from being widely used as a cancer treatment. It has recently become obvious that melittin and/or its conjugates can be used for targeted treatments of various cancer types using melittin as a component of nanoparticles [245,247] or for gene therapy [219].

#### Melittin Expression Hosts

- Melittin Bacterial expression systems

Melittin is easily degraded by proteases in bacterial environments. As a result, direct expression in prokaryotic systems is unfeasible; thus, it is typically produced as a fusion protein with a different protein tag. However, when combined with target peptides, melittin demonstrated a low level of toxicity, so it is important that the fused toxin contains a cleavable linker to release melittin following protease cleavage. Melittin has been expressed and fused with a GST tag to protect it from intracellular protease cleavage and stabilize the structure. Zhou et al. produced recombinant melittin in *E. coli* as a GST-fused protein and obtained an active protein with important antibacterial activity against *E. coli*, *Staphylococcus pasteuri*, and *Bacillus pumilus* [248]. Several works described the use of GST tags in melittin production in *E. coli*: a plasmid called pJB-HTS-MET has been designed to express in *E. coli* Rosetta strain melittin carrying GST and His tags [249]; Shi et al. used the expression vector pGEX-4T-2 to express recombinant melittin, but the fusion protein was not wholly soluble because most of the expressed protein (about 60%) is trapped into inclusion bodies [250]. The melittin gene was bound to a soluble trimer of sTRAIL (tumor necrosis factor-related apoptosis-inducing ligand) and expressed in *E. coli* [224]. The fusion protein containing the SUMO tag was readily purified and demonstrated heightened anticancer efficacy against K562 leukemia cells and HepG2 liver carcinoma cells. The inclusion of the SUMO tag significantly facilitated the production of soluble fusion protein (85% of the total protein content) if compared with the expression of the fusion protein sTRAIL–melittin using the pET28a vector, which tends to promote protein accumulation in inclusion bodies.

To express a soluble form of melittin, it could also be coupled with other functional proteins. Recombinant melittin fused with gelonin (rGEL-Mel) was expressed in *E. coli*, and the fusion chimera showed enhanced cytotoxicity and cellular internalization rates if compared to the proteins alone [222]. By fusing MET with mutant human interleukin 2 (MhIL-2) with the vector pET-15b, Liu et al. successfully produced the novel fusion protein MET-MhIL-2 in *E. coli*. 

In vivo, tumor development was suppressed by this fusion protein, which demonstrated the functional activities of IL-2 and MET [223]. Using the SUMO technology, Chen et al. obtained about 25 mg of melittin protein from 1 L of *E. coli* fermentation culture. The authors constructed a pET-3c-SUMO-melittin plasmid, the SUMO-melittin chimera was cleaved by the SUMO protease, and the protein was purified by Ni+2-NTA chromatography [251].

The objective of cancer immunotherapy is to enhance the immune response against tumors by improving immune cell activity and inversing tumor-induced immunosuppression. Numerous immunotherapy approaches, such as cytokines, tumor vaccines, and monoclonal antibodies, have demonstrated promising therapeutic effects in both cancer patients and animal models. A dual-functional fusion protein, melittin-MIL-2, comprising melittin and a variant of human interleukin 2 with mutations at positions Arg88 and Ala125, was produced in *E. coli* by transforming bacterial cells with the plasmid pET15b-melittin-MhIL-2 [222]. The fusion protein demonstrated the ability to inhibit the growth of SKOV3 human ovarian cancer cells in vitro. Additionally, it displayed inhibitory effects on tumor growth in vivo, as evidenced by its efficacy in human SMMC-7721 cancer cells (liver), A549 cancer cells (lung), and SKOV3 cancer cells (ovarian) cancer xenograft models [252]. 

**Figure 11:**
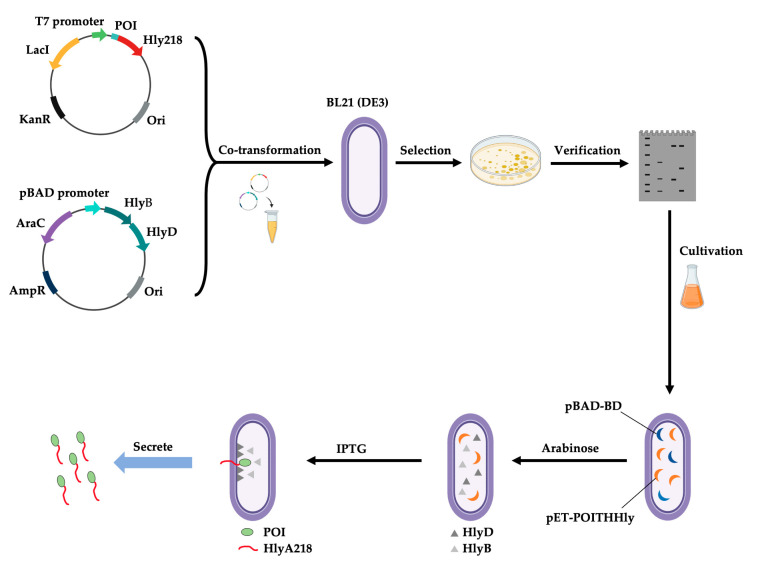
Scheme of plasmid construction, expression strain selection, and protein secretion. The co-transformation of the expression plasmid and accessory plasmid into BL21 (DE3) was followed by LB-Amp + Kan agar plate selection. Subsequent induction with arabinose and IPTG leads to the secretion of the protein of interest (POI) into the supernatant. The figure has been adapted from [253].

The *E. coli* hemolysin secretion system is a versatile method for secreting a diverse range of heterologous fusion proteins into the extracellular medium because it forms no periplasmic intermediates, making it a suitable option for the construction of an exogenous protein secretory production platform. Melittin was successfully produced in *E. coli* for the first time using an innovative production platform called THHly, which relies on the HlyA secretion system. In this process, the MET gene was fused with the signal sequence of HlyA (C-terminal), allowing its secretion to be mediated through the accessory proteins hemolysin B (HlyB) and hemolysin D (HlyD) [253]. This method was recently used for the recombinant production of melittin and other anti-microbial peptides (Figure 11). 

An increasingly common host that produces recombinant proteins is *Bacillus subtilis* because it has effective secretory mechanisms that enable the release of proteins into the growth medium, making it easier to isolate and purify them [254]. Many expression systems have been described for *B. subtilis*, with several inducers such as IPTG, xylose, tetracycline, and T7 polymerase. Due to their quick growth rate, these systems also have a brief fermentation period. These methods have been used to biosynthesize several heterologous proteins while avoiding inclusion bodies and unfeasible fermentation issues related to the growth procedures of *E. coli* and *P. pastoris*, respectively. The host strain, *B. subtilis WB700*, has the advantage of minimizing the degradation of secreted proteins because of the lack of several proteases. This strain has been used to produce a Cecropin A-melittin mutant using the EDDIE fusion technology [254]. 

The first melittin-based recombinant immunotoxin was described in 1996 by Dunn et al. [230]. The expression of the recombinant scFv-mel gene and the purification of the protein product were obtained in *E. coli* TOPP2 cells with enhanced cytolytic activity. More recently, the *E. coli* expression system was also used to produce the anti-CTLA-4-scFv-melittin immunotoxin [229] and the C1–melittin immunotoxin [226].

- Melittin Yeasts expression systems

As previously described, some disadvantages of *E. coli* utilization for recombinant melittin production include bacterial LPS contamination and product toxicity for recombinant *E. coli* [255]. This is one of the reasons why yeasts are the preferred hosts for recombinant melittin production. An expression vector using the human urokinase-type plasminogen activator (uPA)1_43 DNA sequence was employed for the synthesis of the recombinant human uPA1 43-melittin (rhuPA1 43-melittin) chimera in *P. pastoris* and to investigate its antitumor properties against ovarian cancer [220]. The pPICZC plasmid included DNA sequences that encoded the amino acids uPA1-43 and melittin, respectively, and matched their native amino acid sequences. The *P. pastoris* X-33 strain was then transformed using the recombinant vector, and rhuPA(1-43)-melittin was expressed via methanol induction. Another example of *P. pastoris* utilization is the production of the rATF-melittin chimera, in which the melittin sequence is fused with only the amino-terminal fragment (ATF) of uPA, which might benefit from melittin’s anticancer properties as well as ATF’s particular binding to upregulated uPAR on tumor surfaces [221]. 

A pPIC9-Melittin vector was more recently designed and used for efficacious integration into *P. pastoris*; the presence of the alpha-factor signal for secretion, the His4 gene, and the alcohol oxidase 1 promoter (PAOX1) in the vector pPIC9 allowed for the expression of the protein with the use of a minimal medium [256]. 

In this way, due to the secretion signal alpha factor, the recombinant melittin is expressed as a secretory protein and released into the culture medium. When a culture medium devoid of both protein and peptide is used, the total protein content in the supernatant is related to the recombinant peptide because *P. pastoris* does not naturally produce considerable amounts of secreted proteins [257]. Another sequence, His4, produces an enzyme that serves as the primary growth factor for histidine biosynthesis. *P. pastoris* may develop on a culture medium devoid of peptides thanks to HIS4 [258,259]. 

Other examples of melittin chimeras expressed in yeasts are the M-IL-2((88)Arg, (125)Ala) fusion protein, composed of melittin genetically linked to a mutant human interleukin 2((88)Arg, (125)Ala) [235] and the human vascular endothelial growth factor-melittin (VEGF-MEL) fusion protein [233,260].

- Melittin Adenovirus expression systems

In gene therapy, it is necessary to make sure that the expression of therapeutic genes is limited only to the tissue of interest in an effort to increase the treatment index. This is crucial for suicide gene techniques because it can cause substantial toxicity when harmful genes are expressed at low levels in normal tissues, and one promising solution to this issue depends on the capacity to tightly regulate gene expression at the transcriptional level. An example of the application of this strategy is reported in a study from 2005 in which constructs carrying the Mel gene and a-fetoprotein (AFP) promoter (Ad-rAFP-Mel) were used to produce recombinant adenoviruses. Upon transduction via Ad-rAFP-Mel, Mel mRNA was transcribed and expressed in BEL-7402 hepatocellular carcinoma cells, strongly inhibiting their proliferation [227]. 

Similarly, the LAP domain of TGF-ß was fused with melittin, with an MMP2 cleavage site in the middle, to create a recombinant adenovirus that encodes a tumor-activated pro-cytolytic peptide. The melittin-MMP2-LAP recombinant adenovirus can be triggered by MMP2, which releases free melittin to lyse the target cells, according to in vitro tests. Based on in vivo investigations, mice treated with melittin-MMP2-LAP recombinant adenovirus had a B16 tumor volume that was around 70% lower than that of control mice [225]. 

In order to target AFP-positive cancer cells in hypoxic environments, Qian C.Y. et al. successfully developed a cancer-specific oncolytic adenovirus called QG511-HA-melittin. In this construct, the hypoxia-response element (HRE)-AFP promoter is employed to regulate viral E1a expression, specifically targeting AFP-positive cancer cells; additionally, the E1b-55 kDa gene has been removed [219].

- Gene therapy with melittin non-viral vectors

Survivin, an inhibitor of the apoptosis gene family, is upregulated in the majority of cancer tissues but not in healthy ones. According to most recent reports, the activity of the survivin promoter is tumor-specific, making it an excellent option for use in the creation of gene therapy vectors. In a study published by Qu L. in 2014, a non-viral vector (pSURV-Mel) was created to test the anti-cancer effects of the Mel gene in a mouse model of a human HCC xenograft tumor and in HCC cell lines. The plasmid produced melittin in cancer cells, promoting cytotoxicity and also inhibiting the development of xenograft tumors [228].

## 5. Conclusions

Toxins and their fusions (i.e., immunotoxins) can be generated in significant quantities through heterologous expression, allowing the exploration of the biotechnological potential of these bioactive proteins. In particular, recombinant immunotoxins can be designed in various formats of smaller antibody fragments (25–200 kDa) since only the variable regions of the antibody are needed. In other words, immunotoxin design can occur using more compact antibody fragments, focusing attention on variable regions that are crucial for recognition and binding to specific cellular targets. This strategy helps maintain efficacy in targeting desired cells while reducing the overall size of the molecule, potentially improving tissue penetration and speed of clearance from the body, as well as reducing immunogenicity. The ultimate goal is to develop more precise and effective targeted therapies, especially in the context of cancer treatment.

- Prokaryotic hosts

Among the various systems for producing heterologous proteins, *E. coli*, a Gram-negative bacterium, continues to be one of the most attractive hosts. Because of its ability to rapidly grow in a high-density environment in a low-cost medium, its well-defined genetic traits, and the multiplicity of cloning vectors and mutant host strains, *E. coli* offers an efficient and cost-effective method for the rapid and high-yield production of proteins. The use of bacterial hosts in the context of producing recombinant toxins and fusion proteins necessitates either resistance to the toxin or the capability to facilitate the accumulation of the protein of interest (POI) in organelles that spatially separate the produced toxin from its molecular target, potentially preventing unwanted cytotoxic effects within the host cell. This spatial separation can enhance the overall yield of the desired product.

Concerning the production of RITs in *Escherichia coli*, the absence of N-linked glycosylation may lead to differences in the behavior of the expressed monoclonal antibodies compared to those produced in mammalian cells. This can include changes in antibody binding strength, serum persistence, and a possible immunogenic response. In bacteria, mAb-based RITs are incorrectly folded and assembled, necessitating costly multi-step refolding processes, which frequently begin with denatured inclusion bodies.

In summary, the choice of a prokaryotic host for toxins or RIT production involves considerations related to both resistance to the toxin and the cellular localization of the POI. These factors play a crucial role in optimizing the production and guaranteeing increased production of the recombinant immunotoxin.

- Eukaryotic hosts

CHO cells are one of the most commonly used mammalian cell lines for the production of therapeutic proteins, including recombinant toxins. They offer the ability to perform complex post-translational modifications, such as glycosylation, and are suitable for large-scale production. Toxin-resistant CHO cell lines are engineered to be resistant to the toxic effects of the coupled toxin, but the presence of the toxin may still affect cell viability and overall productivity. High toxicity can limit the amount of recombinant toxins and RITs that can be produced before negatively impacting the cells. The yields of RITs in these lines are noted to be low, with an example given of 0.004 g/L for an anti-CD3 single-chain variable fragment (scFv) coupled to a truncated diphtheria toxin. This is in contrast to the higher yields frequently reported for monoclonal antibodies (mAbs), reaching 5 g/L during fed-batch fermentation. *Pichia pastoris* can produce functional mAbs and has been successfully used to produce more than 10 toxins and derivatives with titers of up to 0.770 g/L. However, the final yield of RITs can be frequently affected by the release of proteases.

Plant-based expression systems offer several advantages, including cost-effectiveness, scalability, and the potential for post-translational modifications similar to those in mammalian cells. Some toxins that have been successfully produced in plants include ricin, the cholera toxin B subunit, and diphtheria toxin. Plant-based expression systems offer advantages such as reduced production costs, scalability, and the potential for oral delivery of therapeutic proteins. Identical expression vectors can be used in different plant-based systems, and this allows for the rapid selection of host platforms that are compatible with the properties of a specific protein product. Plants are an ideal expression host for immunotoxins due to their ability to produce both mAbs and toxic proteins on the same platform in a single process. This straightforward approach is possible because plants naturally produce lectins and have evolved to sequester them within intracellular compartments to keep them separate from their target molecules and proteases. Despite the benefits and variety of recombinant proteins produced in plants or plant cells, there are only a few RITs that have been expressed in plant-based systems.

In Table 3, a comprehensive overview of the expression systems used for the production of the described recombinant toxins and immunotoxins is reported, and the final yields are obtained.

In conclusion, each expression system has its own advantages and challenges, and the choice depends on factors such as the nature of the toxin, the required post-translational modifications, scalability, and the ultimate application of the recombinant toxin. Further developments in expression systems would thus benefit from the increase in solubility of the recombinant protein, strict folding accuracy, the decrease in inclusion bodies or aggregates, and the improvement of secretion pathways. Researchers often evaluate multiple expression systems to determine the most suitable one for a particular toxin.

## Figures and Tables

**Table 1:** DT fusion toxins and immunotoxins used for cancer studies.

Host	DT Fusion Protein/Immunotoxin	Target	References
*E. coli*	DAB486IL-2	Tumor cells	[26]
*E. coli Rosetta Gami* (*DE3*)	DAB389IL-2 (Denileukin Diftitox^TM^)	Cutaneous form of T-cell lymphoma	[27]
*P. pastoris*	DAB389-IL2IL2	Human CD25(+) cells (regulatory T cells)	[28]
*C. diphteria*	s-DAB-IL2(V6A)	PD-1 in melanoma cells	[29]
*E. coli BL21* (*DE3*)	DT388-GM-CSF	Acute myeloid leukemia blasts	[30]
*E. coli BLR* (*DE3*)	DT388-IL3	Acute myeloid leukemia blasts	[31]
*E. coli*	DT389-EGF	Human glioblastoma multiforme cells	[32]
*E. coli*	DT389-YP7	Hepatocellular cancer cells	[33]
*P. pastoris*	A-dmDT390-bisFV	T-cell leukemia and autoimmune diseases	[34]
*E. coli*	DT2219	Human CD19 and CD22 receptors in a mouse xenograft model of B-cell leukemia/lymphoma	[35]
*E. coli*	DTAT/DTAT13/DTATEG	Urokinase-type plasminogen activator receptor on human glioblastoma tumors	[36]
*E. coli*	DTEGF13	Pancreatic cancer cells	[37]
*P. pastoris*	DT-antiCCR4	Human CCR4(+) cells	[38]
*E. coli*	DT386-BR2	Tumor cells	[39]
*E. coli*	DT389GCSF	Granulocyte colony-stimulating factor (G-CSF) receptor	[40]
*E. coli*	DL_9_F and DL_2_F	Human ovarian teratocarcinoma	[41]
*E. coli*	DT389-GRP	Small cell lung cancer cells	[42]
*293T cells*	DAB389-IL7	IL-7(+) tumor cells	[43]
*E. coli*	DT390-biTMTP1/DT390-triTMTP1	Highly metastatic tumors	[44]
*E. coli*	DT-SCF	c-kit(+) tumor cells	[45]
*E. coli BL21* (*DER*)	DTIL13	Human glioblastoma multiforme cells	[46]

**Table 2:** Recombinant melittin chimeras and conjugates used in cancer studies.

Host	Recombinant MEL Constructs	Target	References
*Adenovirus*	QG511-HA–melittin	-	[219]
*P. pastoris*	RhuPA1-43–melittin	Hepatocellular carcinoma cells	[220]
*P. pastoris*	rATF–melittin	Ovarian cancer cells	[221]
*E.coli*	Gelonin–melittin	Ovarian cancer cells	[222]
*E. coli*	Melittin–MhIL-2	Tumor cells	[223]
*E. coli*	sTRAIL–melittin	Human ovarian cancer SKOV cells	[224]
*Adenovirus*	MEL-MMP2-LAP	Leukemia cells and liver carcinoma cells	[225]
*E. coli*	C1–melittin immunotoxin	Ovarian cancer cells	[226]
*Adenovirus*	Ad-rAFP-Mel	Hepatocellular carcinoma	[227]
*Adenovirus*	pSURV–Mel (non-viral vector)	Hepatocellular carcinoma	[228]
*E. coli*	anti-CTLA-4-scFv-melittin	Hepatocellular carcinoma	[229]
*E. coli*	scFvK121-melittin	Potential immunosuppressive agent for organ transplant	[230]
*Baculovirus*	scFv-mel-FLAG	KMA(+) tumor cells	[231]
*E. coli*	GST-melittin	Human lymphoblastoid cells	[232]
*P. pastoris*	VEGF165-melittin	-	[233]
*E. coli*	scFv-Mel-Gal4	Hepatocellular carcinoma	[234]
*P. pastoris*	M-IL-2((88)Arg, (125)Ala)	Liver diseases	[235]

**Table 3:** Summary of the expression systems used for production of the recombinant toxins and immunotoxins described and the final yields obtained.

Toxin	Recombinant Toxins	Expression Sistem	Compartmentalization	Yield	Reference	Year
Diphtheria Toxin	Full protein	Bacteria				
		*E. coli* BLR (DE3)	Cytoplasmatic, inclusion bodies	1.84 mg/mL	[31]	2004
		*E. coli* BL21 (DE3) PlysS	-	1.2 mg/mL	[21]	2022
	CRM197	*Corynebacterium diphtheriae*	Secreted	175–250 mg/mL	[16]	1983
		*Bacillus subtilis*	Secreted	7.1 mg/mL	[66]	1999
		*E.coli* BL21AI	Cytoplasmatic, inclusion bodies	7.1 mg/mL	[59]	2011
		*E. coli*	Cytoplasmatic	154 mg/mL	[60]	2016
		*E. coli* B843 (DE3)	Periplasmic	>3 mg/mL	[58]	2017
		*E. coli*	Cytoplasmatic, soluble	106 mg/L	[62]	2017
		*E. coli* ClearColi BL21 (DE3)	Cytoplasmatic, inclusion bodies	196 mg/mL	[54]	2018
		*E. coli*	Cytoplasmatic, soluble	130 mg/mL	[260]	2022
		*E. coli* Shuffle T7	Cytoplasmatic, soluble	150–270 mg/L	[10]	2023
		Yeast				
		*Pichia pastoris*	Secreted	>100 mg/L	[7]	2021
		Bacteria				
Exotoxin A	Full protein	*Pseudomonas aeruginosa,* PAO1	Secreted	-	[107]	2018
	PE38KDEL	*E. coli* BL21 (XDEB)	Cytoplasmatic, inclusion bodies	1 mg/mL	[109]	1991
	PE40-antiCD22	*E. coli*	Cytoplasmatic, inclusion bodies	3 mg/L	[111]	2015
	D2C7-(scdsFv)-PE38KDEL	*E.coli BLR DE3*	Cytoplasmatic, inclusion bodies	30 mg/L	[110]	2017
		Bacteria				
Saporin S6	Full protein	*E. coli strain JA221*	Periplasmic	4 ug/L	[146]	1993
		*E. coli BL21 (DE3) PlysS*	Soluble fraction	1–3 mg/L	[163]	1997
	pET-E176K | pET-K234stop	*E. coli BL21 (DE3)*	Soluble fraction	0.1–0.3 mg/L	[150]	2005
	SAP-Ser255Cys	*E. coli BL21 (DE3) PlysS*	Soluble fraction	2.7 mg/L	[151]	2008
	SapVSAV	*E. coli BL21 (DE3) PlysS*	Cytosolic fraction	-	[152]	2010
	hCASK- SAP | (hCASK)2-SAP	*E. coli Rosetta Gami^TM^ B pLysS(DE3) | BL21 (DE3)*	Soluble fraction	0.5 mg/L	[153]	2015
	CYS- SAP | RGD-SAP	*E. coli BL21 (DE3)*	Soluble fraction	0.6–1.2 mg/L	[155]	2022
		Yeast				
	ATF-Saporin	*Pichia pastoris*	Secreted	3.5 mg/L	[156]	2010
	SAP-antiCD22	*Pichia pastoris GS115*	Secreted	1–2 mg/mL	[111]	2015
	ATF-SAP chimera	*Pichia pastoris GS115*	Secreted	1–5 mg/L *	[164]	2016
			Secreted	3–7 mg/L *	[164]	2016
		Bacteria				
Ricin A chain	Full protein	*E. coli 7118*	Secreted	2–3 mg/L	[189]	1987
	RTA–KDEL | RTA-YQRL	*E. coli JM 109*	Cytoplasmatic, soluble	10 mg/L	[193]	1998
	rRTA | rRTA–YQRL	*E. coli JM 109*	Cytoplasmatic, soluble	10 mg/L	[192]	2004
	RTA-PAP1	*E. coli*	Cytoplasmatic, inclusion bodies	0.22 mg/mL	[202]	2018
	RTAM-PAP1	*E.coli*	Soluble fraction	0.1 mg/mL	[202]	2018
	Ricin-panitumumab	*E. coli BL21 (DE3)*	Cytoplasmatic, inclusion bodies	0.18 mg/mL	[186]	2023
		Bacteria				
Bouganin	Full protein	*E. coli BL21 (DE3)*	Periplasmic	1 mg/L	[207]	2002
	Trastuzumab-deBouganin Conjugate	*E. coli*	-	0.15–0.5 mg/L	[210]	2016
		Bacteria				
Pulchellin	rPAC	*E. coli*	Soluble fraction	3 mg/L	[213]	2005
Melittin	sTRAIL–melittin	*E. coli BL21 (DE3)*	Cytoplasmatic, inclusion bodies	-	[224]	2013
	rGel-Mel	*E. coli BL21 star (DE3)*	Cytoplasmatic, soluble	3 mg/L	[222]	2016
	Cecropin A-melittin mutant	*Bacillus subtilis* WB700	Secreted	159 mg/L	[254]	2017
	GST-MET	*E. coli BL21 (DE3)*	Cytoplasmatic, soluble	3.5 mg/L	[248]	2020
	Full protein	*E. coli BL21 (DE3)*	Cytoplasmatic, soluble	25 mg/L	[251]	2021
		Yeast				
	rhuPA1-43-melittin	*Pichia pastoris*	Secreted	128 mg/L	[220]	2015
	rATF-mellitin	*Pichia pastoris*	Secreted	312 mg/L	[221]	2016
	Cecropin A-melittin mutant	*Pichia pastoris*	Secreted	125 mg/L	[254]	2017

## Data Availability

No original or unpublished data were used.
